# The Challenge of Accounting for the Moderator Effect of Risk Exposure on the Effectiveness of Mindfulness-Based Treatments for Youth

**DOI:** 10.1007/s41042-023-00145-y

**Published:** 2024-01-12

**Authors:** Raquel Nogueira Arjona, Michael Ungar

**Affiliations:** 1 Sussex Addiction Research & Intervention Centre, School of Psychology, University of Sussex, Brighton, United Kingdom; 2 Resilience Research Centre, Dalhousie University, 6420 Coburg Road, PO Box 15000, Halifax, Nova Scotia B3H 4R2 Canada

**Keywords:** Mindfulness-based treatment, Meta-analysis, Risk exposure- Child, Adolescent

## Abstract

Although many positive psychology interventions like mindfulness-based treatments (MBTs) for youth have been used with a wide array of risk exposed populations (children living in poverty, victims of violence, displaced persons, children with disabilities, etc.), the efficacy of MBTs with regard to the level and domain of risk exposure has been largely overlooked. This oversight contributes to a perception of positive psychology as being decontextualized. The purpose of this meta-analysis is to better understand how a young person’s level and domain of risk exposure influences the efficacy of MBTs. Published studies were included if participants were children or adolescents, were RCTs or cluster RCTs, and focused on the effect of an MBT or an MBT component. Specifically, 58 studies were qualitatively analyzed and 42 were quantitatively analyzed. The qualitative analysis evidenced a lack of objectivity in the assessment of risk exposure, with many papers providing very little or no detail on participants’ level or domain of risk exposure (whether threats to psychological wellbeing originate with the individual’s biology or cognitions, or the social, built or natural environment). Our quantitative analysis showed that MBTs significantly improved main outcomes relative to the control conditions, with a small-to-moderate effect size. Where it was possible to identify the level of risk exposure, we found that MBTs positively moderated treatment response, with better response among those participants with a higher level of risk. We conclude that future Positive Psychology research will need to better account for risk exposure if the discipline is to improve our understanding of which populations benefit most from interventions that promote mindfulness.

**Supplementary Information:**

The online version contains supplementary material available at 10.1007/s41042-023-00145-y.

## Introduction

In the last two decades, there has been growing interest in the use of mindfulness-based treatments (MBTs) to improve resilience, manage mental and physical health and promote well-being in youth. MBTs focus on modifying the response of individuals to the distress caused by internal and external risk factors (i.e., factors associated with increased likelihood of negative outcomes) (Kraemer et al., 2005) rather than on modifying the source of the distress itself (Crane et al., 2017). Despite evidence for the efficacy and effectiveness of MBTs, very little is known about the risk profiles of the populations that benefit from these interventions. While most of the research in this field has provided extensive assessment of changes to cognitions and social functioning attributable to MBTs, there has been to date very little discussion of how different risk profiles impact the likelihood that an MBT will produce a positive change in functioning. We do not know how a child’s degree of risk exposure, or how the many domains of risk that originate with a child’s biology, cognitions, social interactions, or exposure to political, economic and environmental threats to development, influence the differential impact of an MBT. This lack of contextualization is not unique to studies of MBTs. Positive Psychology as a discipline has been critiqued for this same absence of attention to the challenges marginalized populations face and the differential impact interventions may have for individuals exposed to varying levels of risk (van Zyl et al., 2023).

The purpose of this systematic review and meta-analysis is, therefore, to better understand how a young person’s level and domain of risk exposure influences the efficacy of MBTs. Specifically, our aims were: 1) to synthesize the effect of MBTs (overall, anxiety, depression, executive functioning, stress, mindfulness, and functioning and wellbeing outcomes) on youth; 2) to identify the risk factors reported in randomized controlled trials (RCTs) with youth and document when and where risk factors are not sufficiently documented; and 3) to establish the differential impact (degree of effectiveness) of MBTs depending on the level and domain of risk exposure for youth populations. Thus, our unique focus is to critically analyze how studies of MBTs approach the assessment of individual, social, built or natural risk factors and whether accounting for risk challenges assumptions about who benefits from participation in these treatments (for a critical analysis of what constitutes a mindfulness practice-a debate beyond the scope of this paper- see Van Dam et al. [2018]).

Though the problem of lack of contextualization was common to many of the studies we selected for review, we were still able to group published works into four broad risk domains: normative risk (any level of stress exposure that does not go beyond usual frustrations or conventional stress, e.g. exams), individual risks (mental health and/or physical health issues), environmental risk (atypical exposure to adversity caused by the social, built and natural environments), or a combination of both individual and environmental risk. Our choice of domains reflects a qualitative sort of the categories used to describe risk in the studies we reviewed, informed by a conceptualization of risk as occurring across nested biopsychosocial and socioecological systems (Ungar, 2021).

In this study, we approach risk exposure from two perspectives inspired by the cumulative risk literature (Jaffee et al., 2007). First, domain of risk was categorized following a socioecological framework (Ungar, 2018) as individual physical or psychological issues (e.g. interpersonal trauma) (Shirk et al., 2014), disadvantaged social, built or natural environments (e.g. low socio-economic status) (Lo et al., 2018) or a combination of individual and environmental domains of risk (e.g. war and mental health challenges) (Gordon et al., 2008). Second, studies were grouped by the participants’ level of risk exposure (low, medium and high) (Ungar & Theron, 2020). Though MBTs have been used with young people exposed to diverse domains and levels of risks, most studies report very few details regarding the quality or quantity of the risk exposure experienced by their intervention population. As a consequence, our characterization of risk factors in this study is relatively simplistic despite the field of risk science being very well-developed. Where details about risk were scarce or under-developed in the papers we reviewed, we based on categorization on what research with similar populations in similar contexts has shown about the nature of the risks individuals experience. We note that this lack of risk specificity has occurred in the field of Positive Psychology despite the rapid proliferation of MBTs globally.

The fact that we struggled to know whether participants of an MBT were experiencing any kind of risk at all suggests the need for caution when interpreting reports on the efficacy of these treatments. At a time when researches are being called upon to account for systemic and structural factors that affect wellbeing (e.g., sexism, homophobia, racism, poverty, the climate emergency, community violence, etc.), it is disturbing that so few of the studies we located for this review describe the risk profiles of their participants in more than superficial ways. Indeed, without this detail, we may fail to explain why a particular intervention with a particular population under stress (or a combination of stressors) mediates or moderates risk exposure and produces a desired positive outcome (Dimidjian & Segal, 2015).

Previous meta-analyses summarized the effect of MBTs for youth on various outcomes (e.g. cognitions, academic performance, socio-emotional outcomes) by approaching risk exposure in different ways. For example, three meta-analyses reported small-to-moderate overall effects (g=0.23–0.40) with samples experiencing normative risk (i.e., students not selected on the basis of their risk exposure) (Carsley et al. 2018; Maynard et al., 2017; Zenner et al., 2014). Three meta-analyses reported small-to-moderate overall effects (effect sizes=0.19-0.32) (Dunning et al., 2019; Klingbeil et al., 2017; Zoogman et al., 2015) for participants under any domain of risk (i.e., with any risk profile, though those risk profiles were not described in any detail). Two meta-analyses reported moderate overall effects (g=0.57–0.62) (Borquist-Conlon et al., 2019; Chimiklis et al., 2018) on at-risk participants experiencing psychological problems (i.e., selected based on a single domain or narrow definition of risk exposure). Thus, to date, meta-analyses focused on at-risk samples reported larger treatment effects.

Looking beyond the positive psychology literature or studies of MBTs, a recurrent finding is that children exposed to cumulative risk factors (whether the total number of factors or the total number of domains) compared to single risk exposures, present more concurrent and long-term developmental sequelae (Evans et al., 2013). For example, there is evidence of the negative impact of cumulative exposure to adversities and the interaction between specific adversities on health outcomes (Ungar & Perry, 2012). Thus, the socioecological context that exposes a child to multiple forms of risk plays a relevant role both in the development of negative outcomes and in the interaction between protective factors and processes, including those which are the focus of MBTs. However, the moderator effect of the level of risk exposure which has been a cornerstone of studies in fields like risk and resilience, developmental origins of health and disease, and adverse childhood experiences, has been scarcely studied in efficacy trials of MBTs, even with randomized samples. At best, studies of MBTs have found that the level of individual psychological risk moderated the response to an MBT, with children with higher scores in psychological symptoms responding better to treatment than children with lower scores (e.g. anxious and depressive children [Blake et al., 2018]; children with poor executive functioning [Flook et al., 2010]). Evidence of patterns like this within the environmental domain of risk point towards similar conclusions but the number of studies reporting detailed assessments of social and environmental risk exposure in MBTs is remarkably small. Among the few exceptions are Lo et al. (2018) who found that MBT was more effective for children facing higher levels of parental-child relationship stress (d=.52-.72) than for children experiencing a lower level of risk (d=.37, attention and behavioral regulation outcomes).

Except Dunning et al.’s (2019) previous meta-analyses that we reviewed included both randomized and non-randomized studies. Therefore, one of our aims was to overcome this limitation and to synthesize the effect of MBTs on different outcomes, incorporating newly published studies and including only RCTs. We classified the results by the main reported outcome and by type of outcome (that is, anxiety symptoms, depression symptoms, executive functioning, stress, mindfulness, and functioning and wellbeing outcomes). We hypothesize that MBTs (regardless of the risk profile) will improve overall and specific outcomes with a small to moderate effect size.

Next, to investigate the relative effect of these MBTs according to level of risk (low, medium, high), and domain of risk (normative, health/mental health, environmental, combination of environmental and health/mental health), we hypothesized that MBTs would have a differential impact on young people at high and low levels of risk, with MBTs being most helpful to young people experiencing the most risk (Ungar, 2017). To reiterate, while previous meta-analyses explored the effectiveness of MBTs for children and adolescents, (Dunning et al., 2019) none have analyzed the effect of the level and domain of risk exposure on outcomes. When level of risk of participants is assessed rather than included as a control variable (i.e., the population as a whole is assumed to be homogeneous because of a single categorical variable like a physical disorder or race), response to an MBT may change depending on the level of risk or its severity found within the sample. We also explored the effect of mean age, number of sessions, percentage of females, and risk of bias, based on previous findings showing inconclusive results about each factor’s moderation effect on MBTs.

## Method

We followed preferred Reporting Items for Systematic Reviews and Meta-Analyses (PRISMA) standards (Moher et al., 2009) to conduct this study. This review was registered with PROSPERO [CRD42019124747].

### Eligibility Criteria

Studies were included if: 1) participants were children and adolescents; 2) they were published in an English peer-reviewed journal; 3) they were randomized control trials (RCTs) or cluster RCTs, and 4) the effect of a MBT or MBT component (e.g. breathing meditation) was compared to no intervention, waitlist, or an active control condition (e.g. health education program). Interventions were protocolized programs, including individual, small group, large group or family-centered interventions. Variations in the format (length, frequency of contact, duration) did not hinder inclusion. Where more than one paper relating to the same sample was identified, these were analyzed in combination. When more than one intervention was tested, only data related to the MBT and the control group were considered. Only children’s scores were considered from reliable and valid outcome measures, adapted to, or developed for, children and adolescents.

### Data Extraction

For studies meeting inclusion criteria, the following data were extracted: sample size; means and standard deviations (pre- and post- intervention); first author; year of publication; inclusion and exclusion criteria; sample characteristics, including mean age and standard deviation, number of participants per condition (intention to treat sample was preferred over completers sample), % females; intervention characteristics, including type of intervention (e.g. mindfulness-based stress reduction), number of sessions, frequency and duration in weeks (home practice was not included); control group conditions (no intervention, waitlist, active); main hypotheses; outcome measures, including name of the measure, variables assessed; assessment times (pre, post, and follow-ups only if there were both groups included).

When outcome data were incomplete or not reported, the first author contacted the authors of the study to request this information. Studies with non-reported data after request were only included in the qualitative analysis.

### Risk of Bias in Individual Studies

Methodological quality and risk of bias were assessed using criteria based on the Cochrane Collaboration ‘Risk of Bias’ tool (J. Higgins et al., 2019) and the Jadad scale (Jadad et al., 1996). Studies were assessed in relation to: a) adequate allocation sequence generation, b) adequate allocation concealment, c) blinding of main outcome assessments, d) description of withdrawals/drop-outs, e) intention-to-treat analysis is performed or there are no drop-outs, f) sample size is based on an adequate power analysis, g) groups are similar on prognostic indicators at baseline or adjustments were made to correct for baseline imbalance, h) diagnostic assessment was conducted by a professional. Risk of bias for each criterion has been reported individually as low, high or unclear risk of bias.

### Statistical Analyses

#### Calculation of Effect Sizes

The Metafor package in R version 3.5.3 was used to calculate individual and pooled effect sizes. For each comparison between an MBT and control group, effect size was computed using Hedges’ g. This statistic was calculated, first, by computing the mean change scores (M_post_−M_pre_) between the intervention and control groups. The mean change scores were divided by the pooled estimates of the intervention and control standard deviations at pre-intervention (SD_pre_) and corrected for positive bias (Cp) as an adjustment to address small sample sizes (Hedges and Olkin, 1985) The 95% confidence interval for the effect size is reported. Effect sizes of 0.2, 0.5 and 0.8 are interpreted as small, moderate and large effect sizes respectively (Cohen, 2013). As considerable heterogeneity among studies was expected, a random-effects model was used. We assume that the studies are a random sample of possible effect sizes and that the true effect size fluctuates based on intervention or population characteristics. Thus, the primary outcomes may differ as a function of the domain/level of risk exposure, intervention length or type of control group. A random-effect model generates wider confidence intervals than a fixed-effects model and, therefore, is more conservative (Borenstein et al., 2010).

Primary outcomes identified as such by the studies were used to calculate the overall effect. These outcomes were classified based into: anxiety symptoms, depression symptoms, executive functioning, stress, mindfulness, and functioning and wellbeing outcomes. To address dependency among effect sizes we followed recommendations by Gleser and Olkin (2009) using the MAd package. If a total scale was not reported, a correlation of r=.6 was assumed between subscales of a single measure for aggregation. A correlation of r=.5 was assumed when aggregating different outcome measures within a given study (see Wampold et al. (1997) for a rationale). A forest plot of pre-post between-group effect sizes was produced for each study´s main outcomes.

#### Test of Homogeneity

Heterogeneity of effect sizes was calculated using Q and I^2^ statistics. A significant Q statistic (p<=0.05) indicated significant heterogeneity, suggesting that one or more variables moderated the observed effect size. The I^2^ statistic was used to estimate the percentage of heterogeneity across the studies not attributable to random sample error alone. A value of 25%, 50% and 75% indicate low, moderate and high degrees of heterogeneity, respectively (Higgins & Thompson, 2002).

#### Moderator Analyses

Two independent judges (PhDs) classified the studies by domain of risk using the information provided in the inclusion/exclusion criteria and the description of the sample. Categories were: normative risk, non-normative physical and/or mental health challenges, non-normative environmental risks or a combination of non-normative individual and environmental risks (see Table S1). There was substantial agreement between both raters (k=0.87) according to Landis and Koch (1977) categories (Cohen´s kappa coefficient: <0.61 moderate, 0.61-0.80 substantial and 0.81-1 almost perfect agreement). The judges discussed disagreements on the type of risk until they reached consensus. Following the same conceptual framework as other studies (Evans et al., 2013), we created a cross-domain cumulative risk score to assess level of risk exposure (0=normative risk, 1=one domain of risk, individual or environmental, 2=two domains of risk, individual and environmental).

Two sets of moderator analyses on treatment effects were conducted for continuous and categorical moderators. First, we conducted meta-regression using restricted maximum likelihood (REML) estimation with continuous moderators (level of risk, mean age, number of sessions, percentage of females, and risk of bias). We lowered the alpha threshold for significance to P<.01 (α=.05/5). Second, we conducted subgroup analysis with the categorical variable domain of risk.

#### Publication Bias

To assess publication bias, we examined the funnel plot of the primary outcomes depicting effect estimates against their corresponding standard errors. We tested funnel plot asymmetry by conducting Egger’s regression (Egger et al., 1997).

## Results

### Study Selection

**Fig. 1:**
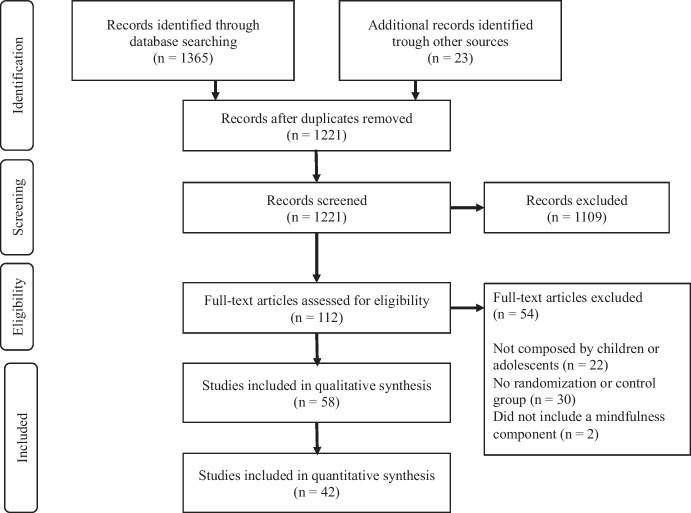
PRISMA flow diagram of the search results

Figure 1 shows a PRISMA flow diagram of the search results. Search parameters specified studies published between 2000 and 2019, in English. We purposely did not select studies published during the COVID-19 pandemic as the overwhelming risk posed by the pandemic would be expected to obscure other risk exposures such as economic and social marginalization (future studies of MBTs post-pandemic should, therefore, be the subject of next phase meta-analyses). Characteristics of the studies detailing the different MBTs involved are presented in Table 1.

**Table 1:** Summary of studies included in quantitative and qualitative analyses

First author (year)	% F	Mean age (SD)	Intervention (n)	Session length, n sessions, n weeks	Control group (n)	Meas.^4^
Barnes et al. (2001)	45.71	16.55 (1.1)	TM program (17)	15m/day, 40 s,8 w	Health education (18)	Pre, post
Barnes et al. (2003)	28.88	16.11(1.34)	TM program (25)	30 m/day, 16 w.	Health education (20)	Pre, post
Barnes et al. (2004a)	46.57	12.3 (0.6)	Meditation (34)	14 m/day, 12 w.	Health education (39)	Pre, post
Barnes et al. (2004b)	37	16.15 (1.35)	TM program (50)	30 m/day, 16 w.	Health education (50)	Pre, post, 4-mth
Napoli et al. (2005)	47.36	N.R. (N.R.)	AAP (114)	45 m, 12 s., 24 w.	Reading (114)	Pre, post
Barnes et al. (2008)	42.42	15.20 (0.84)	BAM (20)	10 m., 60 s., 12 w.	Health education (46)	Pre, post
Gordon et al. (2008)	75.6	16.3 (N.R.)	Mind-body (41)	120 m, 12 s., 6 w.	Wait-list (41)	Pre, post
Biegel et al. (2009)	73.5	15.35 (1.20)	MBSR+TAU (50)	120 m, 8 s., 8 w.	TAU (52)	Pre, post, 3-mth
Catani et al. (2009)	45.16	11.93 (2.0)	KIDNET(16)	90 m, 6 s., 2 w.	MED-RELAX (15)	Pre, post, 6-mth
Barnes et al. (2010)	61.40	15.05 (0.72)	BAM (56)	10 m, N.R., N.R.	Health education (58)	Pre, post
Flook ((Liehr & Diaz, 2010)2010)	54.69	8.23 (0.66)	MAPs (32)	30 m, 16 s., 8 w.	Silent reading (32)	Pre, post
Liehr (2010)	29	9.5 (1.6)	MI (9)	15 m., 10 s., 2 w.	Health education (9)	Pre, post
Mendelson et al. (2010)	60.8	10.08 (0.7)	MBSR (51)	45 m, 48 s., 12 w.	Wait-list (46)	Pre, post
Semple et al. (2010)	60	10.48 (1.23)	MBCT-C (13)	90 m, 12 s., 12 w.	Wait-list (12)	Pre, post
Terjestam et al. (2010)	50.64	13.14 (N.R.)	Quigong (85)	25 m, 16 s., 8 w.	School-as-usual (71)	Pre, post
Wright et al. (2011)	63.29	15.05 (0.76)	BAM (35)	10 m., 60 s., 12 w.	Health education (44)	Pre, post, 3-mth
Gregoski et al. (2011)	60.82	15.09 (0.77)	BAM (53)	10 m., 60 s., 12 w.	Health education (44)	Pre, post
White (2012)	100	9.9 (0.72)	MA (70)	60 m., 8 s., 8 w.	Wait-list (85)	Pre, post
Evans-Chase (2013)	0	18 (1.30)	MP3-MM (29)	60 m, 8 s., 8 w.	MP3-PMR (30)	Pre, post
Leonard et al. (2013)	0	17.4 (0.71)	PS (147)	75 m., 10 s.,3-5 w.	C-P Intervention (117)	Pre, 3-mth
Jastrowski Mano et al. (2013)	83.33	14.16 (1.90)	MBSR (4)	90 m., 6 s., 6 w.	Psychoeducation (2)	Pre, post, 12-w
Sibinga et al. (2013)	0	12.5 (N.R.)	MBSR (22)	50 m., 12 s., 12 w.	Health education (19)	Pre, post
Britton et al. (2014)	45.54	11.79 (0.41)	ICP (52)	3-12 m., 30 s.,6 w.	African History (48)	Pre, post
Parker et al. (2014)	57.65	10.09 (0.51)	Master Mind (71)	15 m., 20 s., 4 w.	Wait-list (40)	Pre, post
Raes et al. (2014)	63.61	15.40 (1.2)	MBCT+SR (201)	100 m, 8 s., 8 w.	School-as-usual (207)	Pre, post, 6-mth
Shirk et al. (2014)	83.72	15.30 (1.53)	MCBT (20)	N.R., 12 s., 12 w.	TAU (23)	Pre, post
Sibinga et al. (2014)	80	15.0 (N.R.)	MBSR (26)	120 m., 8 s., 8 w.	Health education (17)	Pre, post
van de Weijer-Bergsma et al. (2014)	55	9.92 (0.92)	MindfulKids (95)	30 m., 12 s., 6 w.	Wait-list (104)	Pre, post, 2-mth
Atkinson and Wade (2015)	100	15.7 (0.77)	MBI (138)	3 s., 3 w.	School-as-usual (101)	Pre,post,1,6-mth
Flook et al. (2015)	50	4.67 (0.27)	KC (30)	30 m, 24 s., 12 w.	Wait-list (38)	Pre, post
Himelstein et al. (2015)	0	16.45 (N.R.)	MBSUT (14)	90 m.,12 s., 12 w.	TAU (13)	Pre, post
Jee et al. (2015)	45.23	16.8 (1.8)	MBSR (21)	120 m., 8 s., 8 w.	No intervention (21)	Pre, post
Ricarte et al. (2015)	45.55	8.9 (1.98)	MEITP (45)	15 m., 30 s., 6 w.	Wait-list (45)	Pre, post
Schonert-Reichl et al. (2015)	44	10.24 (0.53)	MindUP (48)	50 m., 12 s., 12 w.	BAU (51)	Pre, post
Tan and Martin (2015)	75	15.40 (1.55)	TAU+TAM (46)	60 m., 5 s., 5 w.	TAU (45)	Pre, post, 3-mth
Blake et al. (2016)	59.34	14.48 (0.95)	Sleep SENSE (63)	90 m., 7 s., 7 w.	Study SENSE (60)	Pre, post
Bluth et al. (2016)	39	17 (1.3)	BREATHE (14)	50 m., 11 s., 11 w.	Subst. prevention (13)	Pre, post
Daly et al. (2016)	100	15.52 (1.12)	MEI (14)	90 m., 6 s., 6 w.	TAU (23)	Pre, post
Johnson et al. (2016)	47.7	13.63 (0.43)	Dot be (132)	60 m., 8 s., 8 w.	School-as-usual (176)	Pre, post, 3-mth
Poehlmann et al. (2015)	49	3.91 (0.52)	KC (15)	30 m., 24 s., 12 w.	TAU (14)	Pre, post, 3-mth
Quach et al. (2016)	62	13.18 (0.72)	MBSR (61)	45 m., 8 s., 4 w.	Wait-list (57)	Pre, post
Sibinga et al. (2016)	50.33	N.R (N.R.)	MBSR (141)	50 m, 12 s., 12 w.	Health education (159)	Pre, post
Felver et al. (2017)	57	11.08 (1)	MFSR (24)	90 m., 8 s., 8 w.	Wait-list (23)	Pre, post
Johnson et al. (2017)	45.4	13.44 (0.33)	Dot be (169)	60 m., 9 s., 9 w.	School-as-usual (151)	Pre, post, 6,12mth
Shomaker et al. (2017), Shomaker et al. (2019a)	100	14.99 (1.57)	BREATHE (17)	60 m., 6 s., 6 w.	Blues program (16)	Pre, post, 6-mth
Mak et al. (2018)	42.85	9.25 (3.03)	MiYoga (21)	90 m., 6 s., 8 w.	Wait-list (21)	Pre, post
Lo et al. (2018)	44.11	6.50 (0.83)	FMBI (51)	60 m., 8 s., 12 w.	Wait-list (51)	Pre, post
Webb et al. (2018)	45.83	18.71 (2.31)	MBSR (38)	N.R., 9 s., N.R. w.	Health education (34)	Pre, post, 3-mth
Lawler et al. (2019)	60.4	7.79 (1.6)	MT (38)	60 m., 6 s., 6 w.	No intervention (33)	Pre, post
Lu et al. (2019)	25.4	11.7 (0.6)	MBT (30)	60 m, 8 s., 8 w.	Homework (33)	Pre, post
Patton et al. (2019)	62	14.99 (0.66)	CBT+MM (130)	40 m., 3 s.	No intervention (133)	Pre, post, 3,6-mth
Shomaker et al. (2019b)	55.55	14.21 (1.55)	BREATHE (29)	60 m., 6 s., 6 w.	Health education (25)	Pre, post, 6-mth
Vohra et al. (2019)	40.7	14.2 (1.4)	MBSR + TAU (45)	120 m., 8 s., 10 w.	TAU (40)	Pre, Post, 1-3-mth

*F* female, *SD* Standard deviation, *n* intent to treat sample size, *m* minutes, *s* sessions, *w* weeks, *Meas.* Measurements, *TM* Trascendental Meditation, *N.R.* Not reported, *AAP* Attention Academy Program, *BAM* Breathing awareness meditation, *Mind-body* Mind-body skills group program, *MBSR* Mindfulness Based Stress Reduction, *TAU* Treatment as usual, *KIDNET* Narrative Exposure Therapy for children, *MED-RELAX* meditation-relaxation protocol, *MAPs* Mindful awareness practices, *MI* Mindfulness intervention by Mindful Schools, *MBCT-C* Mindfulness-based cognitive therapy for children, *Quigong* meditative movement exercise, *MA* Mindful Awareness for Girls through Yoga program, *MP3-MM* MP3-delivered MM, *MP3-PMR* MP3-delivered Progressive Muscle Relaxation, *PS* Power Source, *C-P Intervention* Cognitive-perception intervention, *ICP* Meditation according to Roth’s Integrative Contemplative Pedagogy, *MBCT+SR* Mindfulness-based cognitive therapy and mindfulness-based stress reduction, *MindfulKids* program modeled after MBSR and MBCT, *MBI* Mindfulness-based intervention, *KC* Kindness Curriculum, *MBSUT* Mindfulness-based substance abuse treatment, *MEITP* Mindfulness Emotional Intelligence Training Program, *MindUP* Mindfulness-based social-emotional learning program, *BAU* Business as usual social responsibility program, *TAM* Taming the Adolescent mind, *Sleep SENSE* Cognitive-behavioral and mindfulness-based group sleep intervention, *Study SENSE* Study skills educational program, *BREATHE* Learning BREATHE program, *Subst. prevention* Substance abuse prevention, *MEI* Mindful eating intervention, *Dot be* Mindfulness-based cognitive therapy/Mindfulness Based Stress Reduction, *MFSR* Mindful Family Stress Reduction, *MiYoga* mindfulness- based movement programme, *FMBI* family-based mindfulness intervention, *MM* Mindfulness training, *MBT* Mindfulness-based training, *MM* Mindful Meditation

^1^Studies corresponding to the same trial.

^2^Follow-ups involving both experimental and control groups.

^3^Summary for Evans-Chase (2013) and Evans-Chase et al. (2015).

^4^Summary for Blake, et al. (2016), Blake, Snoep et al. (2017), Blake, Schwartz et al. (2017), Blake et al. (2018).

### Risk of Bias

**Fig. 2:**
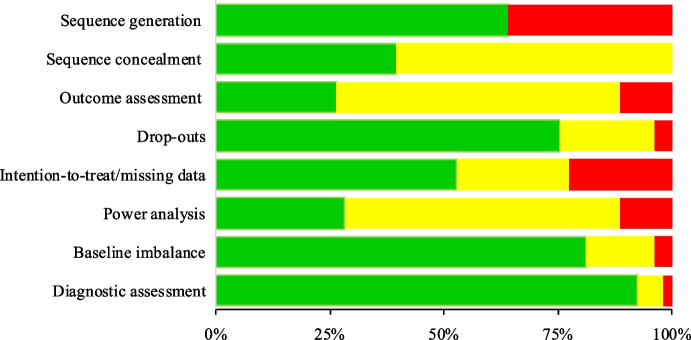
Risk of bias ratings

Risk of bias ratings are presented in Figure 2 and Table S2. The methodological quality was highly variable, with an improvement among the most recent publications (Table S2). Only 39.62% of the studies effectively concealed the allocation sequence, although 64.15% adequately generated it. Only 26.41% of the studies adequately blinded outcome assessments. Only 28.30% of the studies reported power analysis. Most of the studies (75.47%) adequately analyzed dropouts, although only 52.83% reported adequately intention-to-treat analysis or handled missing data. Most of the studies (81.13%) compared samples with similar prognostic indicators at baseline or corrected baseline invariance. In most cases (92.45%) diagnostic assessments were conducted by a professional, or were not necessary.

### Qualitative Synthesis

#### Study Characteristics

Sixteen authors were contacted to request additional information. A sample of 58 studies was included in the qualitative analysis and a sample of 42 studies was included in the quantitative analyses. Table S1 summarizes the main hypothesis, outcome variables and instruments used for each study.

#### Domains of Risk Exposure

According to the evidence provided, 28.07% of studies used samples experiencing normative risk, 26.31% non-normative environmental risks, 29.82% non-normative physical and/or mental health risk, and 15.78% a combination of the latter two domains.

Studies showed evidence of normative risks by reporting sample descriptors (100%; “Middle-school students”), absence of non-normative environmental risks (43.65%; “Reported no recent family stress”) or absence of non-normative physical and/or mental health risks (18.75%; “No hypertensive”).

Studies showed evidence of non-normative environmental risks by reporting sample descriptors (84.61%; “Postwar zone”), percentages of demographic and social indicators (46.15%; “% affected by traumatic experiences”) or outcomes of standardized scales/interviews (23.07%; “Hollingshead's Social Economics Status Scale”).

Studies showed evidence of non-normative physical health risks by reporting patient/other professional’s reports (18.18%; “Referred by educational psychologist as having reading difficulties”), outcomes of standardized scales/classification systems on physical health risk (18.18%; “Pain Frequency-Severity Duration scale”) or values on physiological variables (81.81%; “Systolic blood pressure”). Studies showed evidence on non-normative physical health protective factors by reporting the absence of a risk factor (“Absence of a physical health risk factor”).

Studies showed evidence of non-normative mental health risk by reporting sample descriptors (10%; “Residents of CASA House”), or outcomes of standardized scales/classification systems on mental health risk (90%; “DSM-IV”).

### Quantitative Synthesis

#### Overall and Outcome-Specific Treatment Effect

Table 2 shows overall and outcome-specific effect sizes for all 42 studies (three studies reported on the same sample and were analyzed together). Forty studies including N=4024 participants reported at least one main outcome (see section 5.3.1) and compared an MBT group (N=2003) with a control group (N=2021). The overall effect size at post-intervention for the main outcome was small, with greater outcome improvements following MBT (g=0.33; 95% confidence interval [CI] 0.14 to 0.51; Z=3.48; P<.001). The Figure S1 represents a forest plot depicting individual effect sizes and weighted means. Between-study variation was significant indicating substantial heterogeneity between studies (Q_(39)_=250.30, P=.000, I^2^=89.51%). A funnel plot representing individual effects against their standard error is depicted in Figure S2. Egger’s regression test for funnel plot asymmetry was far from statistical significance, z=1.36, p=.175, indicating that the result of the meta-analysis is not likely to be overestimated by publication bias.

**Table 2:** Effect size analysis of RCT studies examining the efficacy of MBTs (overall and per type of outcome)

Outcome	k	Hedge’s g	SE	z	95% CI	p_1_	I^2^ (%)	Q	df	p_2_
Overall	40	0.33	0.09	3.48	0.14 to 0.51	.000	89.51	250.30	39	.000
Anxiety	14	0.15	0.07	2.11	0.01 to 0.30	.035	41.04	20.19	13	.090
Depression	15	0.22	0.08	2.60	0.05 to 0.39	.009	64.56	39.95	14	.009
Executive functioning	12	0.29	0.13	2.22	0.03 to 0.54	.030	80.56	61.65	11	.000
Stress	10	0.31	0.21	1.44	-0.11 to 0.72	.149	86.09	49.64	9	.000
Mindfulness	14	0.15	0.11	1.40	-0.06 to 0.36	.162	78.34	45.38	13	.000
Funct. & Well.	8	0.09	0.10	0.86	-0.11 to 0.30	.386	63.23	18.88	7	.026

k = number of effect sizes contributing to estimated effect size in the same row. Positive effect size indicates in favour of MBT, *SE* tandard error of the coefficient, *z* z-score associated with the coefficient value in the same row, *p*_*1*_ p-value associated with the coefficient in the same row, *Q* Q-statistic, *df* degrees of freedom of the Q-test, *p*_*2*_ p-value of the Q-test, *Funct. & Well* Functioning and wellbeing

Effect size estimates for each type of outcome ranged from g=0.15 to 0.29, indicating small effect sizes for anxiety, depression, and executive functioning outcomes favoring the MBTs over control interventions. There was no significant effect for stress, mindfulness, and functioning and wellbeing outcomes. The results for heterogeneity tests for significant suggested high heterogeneity for depression and executive functioning outcomes. In the case of anxiety outcomes, the test for heterogeneity failed to reach statistical significance suggesting that the studies are estimating the same true effect (Table 2).

#### Moderator Variables

The results of the meta-regression and subgroup analyses on the study’s main outcome are provided in Table 3. We found a significant moderation effect for the variable level of risk, suggesting that a higher level of risk tends to yield a larger effect size for the study’s main outcome. We found a statistically significant effect for the variable number of sessions, indicating that a higher number of sessions tends to yield a larger effect size. We found no statistically significant differences in effect sizes associated with the mean age, percentage of females or risk of bias.

**Table 3:** Meta-regression and subgroup analyses of effect MBT versus controls on main outcome

Variable		k	Estimate	SE	z	95% CI	p_1_^b^	Q	df	p_2_
Level of risk	Intercept	40	-0.42	0.26	-1.63	-0.92 to 0.09	.10	9.36	1	.00
	Slope		0.40	0.13	3.06	0.14 to 0.66	.00			
Mean age	Intercept	40	-0.37	0.35	-1.06	-1.07 to 0.32	.29	4.19	1	.04
	Slope		0.05	0.03	2.05	0.00 to 0.11	.04			
No of sessions	Intercept	40	0.10	0.12	0.87	-0.13 to 0.33	.38	7.84	1	.00
	Slope		0.01	0.00	2.80	0.00 to 0.02	.00			
% Females	Intercept	40	0.48	0.27	1.77	-0.05 to 1.01	.07	0.35	1	.55
	Slope		-0.00	0.00	-0.60	-0.01 to 0.01	.55			
Risk of bias	Intercept	40	0.23	0.13	1.73	-0.03 to 0.50	.08	1.21	1	.27
	Slope		0.11	0.10	1.10	-0.08 to 0.30	.27			
Domain of risk	Normative	11	0.04	0.15	0.28	-0.26 to 0.35	.78	10.03	2	0.00
	Individual OR environmental	22	0.29	0.19	1.57	-0.08 to 0.66	.13			
	Individual AND environmental	7	0.88	0.28	3.25	0.33 to 1.42	.00			

k = number of effect sizes contributing to estimated effect size in the same row. Positive effect size indicates in favour of MBT, *SE* standard error of the coefficient, *z* z-score associated with the coefficient value in the same row, *p*_*1*_ p-value associated with the coefficient in the same row. Alpha threshold Bonferroni adjusted to *p* < .0125 in meta-regression; *p* < .05 in subgroup analyses; *Q* result of the Q-test for moderation, *df* degrees of freedom of the Q-test for moderation, *p*_*2*_ p-value of the Q-test for moderation

We also found a significant moderation effect of the variable domain of risk, revealing a large significant effect size in studies with samples experiencing a combination of health/mental health and environmental risks compared to studies with normative samples or samples under health/mental health risk or environmental risks alone, with small non-significant effect sizes.

## Discussion

The overarching aim of this study was to better understand the relative effect of MBTs with children and adolescents exposed to different domains and levels of risk. To our knowledge, this meta-analysis is the first to synthesize and evaluate the moderator effect of the domain and level of risk exposure. As methodological quality varied highly across studies, inferences about the quality of the studies must be interpreted with caution due to the fact that details on the blinding of participants and outcome assessors were largely underreported. Meta-regression analysis suggested that risk of bias did not moderate treatment outcome.

Almost 30% of the studies tested the effect of MBTs for youth under normative risk exposure (any level of conventional stress). Most of these studies did not report evidence of absence of risk and instead described the school setting to illustrate the context where the treatment was delivered. When authors reported evidence of absence of risk they usually focused on the environment rather than individual factors, albeit with very little detail. Most studies with samples that reported individual risk exposure (i.e., mental/physical health issues) used objective indicators such as physiological variables or outcomes from standardized scales. All studies with samples where environmental risk exposure (i.e., social, built or natural) was elevated reported context descriptors. These descriptions, however, lacked validity as studies infrequently recorded percentages of demographic and social indicators or outcomes from standardized scales, preferring instead to assess risk by crude demographic factors like exposure to a single risk or individual characteristic. Thus, there is a lack of objectivity in the assessment of variables belonging to social, built and natural environments in spite of the evidence of their role in the development and maintenance of resilience (Ungar & Theron, 2020). Even more challenging to advancing the field of Positive Psychology, there was almost no mention of risk exposure in relation to issues of social justice, equitable access to resources, or any other information required to contextualize the nature of an MBT intervention and its impact on specific populations of young people. This issue is even more complicated if we consider the problem of compliance. There is little to no mention in these studies of which profile of at-risk youth might struggle with practicing mindfulness and the impact of compliance on the efficacy of different approaches to MBTs. Without more attention to contextual factors, claims that mindfulness interventions improve wellbeing for all youth seem overstated.

Our findings suggest the need for a comprehensive socioecological characterization of risk and protective factors when assessing the effect of MBTs. This would, first, improve our understanding of each sample’s level of risk exposure and, therefore, could help mental health professionals to generalize results or aggregate outcomes at equivalent levels and domains of risk. Second, this would shed light on the moderating effect of risk and protective variables across different systems (biological and psychological systems, social, built and natural environments) and could better inform our understanding of the interplay between these variables in the development and maintenance of mental and physical health. This interplay has been proved to be relevant in previous studies exploring resilience processes. For example, Singh et al.(2019) studied the effect of a school-based prevention program targeting depression in adolescents. The authors did not find a significant effect of the program on depressive symptoms even though there was improvement in socio-emotional skills. However, they found that family involvement positively predicted reductions in depressive outcomes among adolescents following the program.

Given the data available in the published papers we reviewed, it was not possible to consider more sophisticated analyses of risk profiles. Ideally, if Positive Psychology is to merit consideration by populations experiencing more extreme adversity and structural disadvantage, it will need to focus on identifying hidden profiles of risk exposure using techniques like Latent Profile Analysis. LPA and other means of portraying fuller accounts of the contextual variation between the settings where interventions are delivered could help to ensure that treatment is offered in ways that are attuned to people’s experiences of privilege and marginalization.

As predicted, and without much consideration of risk profiles, MBTs still showed significantly improved main outcomes relative to the control conditions, with a larger overall effect size than the one reported by Dunning et al.’s (2019) study (0.33 vs 0.19). This difference might be due to the inclusion of new studies in our meta-analysis that are methodologically more rigorous (Patton et al., 2019; Shomaker, Berman, et al., 2019a, 2019b; Vohra et al., 2019), or to methodological differences. For example, we computed the overall effect with the main reported outcome whereas Dunning et al (2019) did the same by summarizing all outcomes. Our results show that MBTs significantly improved anxiety, depression and executive functioning outcomes relative to the control conditions, with small effect sizes. However, there were no significant differences on treatment effect in stress, or mindfulness and functioning outcomes.

Our results do, however, start the process of better understanding the moderation effects of risk exposure on MBT outcomes. Specifically, our meta-analysis showed that level of risk positively moderated treatment response, with better response among those with a higher level of risk. Even more, those more vulnerable given the interaction between individual and environmental risk factors responded better to treatment than those with one risk factor and those under normative stress. This is consistent with results from RCTs that show that individuals under higher levels of risk exposure respond better to MBTs than individuals under lower levels of risk exposure (Flook et al., 2010; Lo et al., 2018; Semple et al., 2010; van de Weijer-Bergsma et al., 2014). This is also consistent with studies that show that children exposed to significant risk but with adequate access to protective factors (e.g. supportive relationships, experiences of efficacy) show more resilience than rugged individuals with fewer external resources (Ungar, 2013, 2017; Ungar & Theron, 2020). Our results align with the cumulative risk literature (Evans et al., 2013) and with the idea that there are critical developmental windows where early interventions might be crucial for treatment effect (Marín, 2016). Thus, this effect might reflect the fact that the developmental period in which protective processes occur is critical to more vulnerable children. Additionally, our results tentatively suggest that MBTs should be targeted to individuals under higher levels of risk rather than applied universally.

Finally, we did not find a significant moderation effect of age or percentage of females. This lack of significant moderation effect might be due to the way our variables were categorized. For example, Carsley et al. (2018) found a significant moderator effect of age using developmental period as a moderator (i.e. middle childhood, early adolescence, and late adolescence).

One limitation of this study is that our results show high heterogeneity indices. Heterogeneity is usually high in studies summarizing the effect of MBTs probably as a result of the varied nature of the hypothesis, and the wide range of variables and outcomes included, as can be appreciated in the supplemental material (Table S1). We believe that this is an important source of variability that might account for the high H indexes. In spite of this heterogeneity, we retained a high number of studies because we were interested in the differential effect of the treatment from a socioecological perspective. An additional limitation is that we use broad categories to conceptualize risk, therefore it is possible that we are missing details on the impact of more graded levels or domains of risk. However, the number of studies including higher levels of risk is more limited and a more detailed categorization was not possible. By including studies with diverse domains of risk we improved the generalizability of our results and have highlighted the importance of a detailed assessment of individual and environmental risk factors when designing MBTs for specific populations.

## Conclusion

Our results suggest that variables belonging to social, built and natural environments are largely overlooked in studies of MBT, a common critique of Positive Psychology as a whole. A more objective assessment of those variables would help to improve our understanding of the mechanisms of treatment that improve mental health and advance the identification of the most vulnerable individuals that would benefit most from treatment. Future research may wish to consider including detailed risk profiles that capture not only individual level risks (e.g., mental health problems like anxiety and depression), but also risk factors across multiple systemic levels. These include: the microbiome; complex intra and extra-familial relationships; contact with and trust in institutions, whether government, police, or the media; social media use; experiences of equity, diversity and inclusion; and the increasingly important interactions people have with the natural environment and our changing climate. Profiles that capture some, or all, of these aspects of a person’s life are expected to help explain the amount of variance we can expect from an intervention targeted towards optimizing wellbeing and social functioning.

We conclude that when risk profiles are accounted for we are better able to assess which populations might benefit most from a Positive Psychology intervention like MBT. Based on our findings, the universal use of MBTs with populations of young people experiencing normative levels of risk may be an unnecessary waste of resources. Policies and protocols for intervention should take this into consideration.

## Supplementary Information

Below is the link to the electronic supplementary material.

Supplementary materials (DOCX)
