# Supplementary material for: The Challenge of Accounting for the Moderator Effect of Risk Exposure on the Effectiveness of Mindfulness-Based Treatments for Youth
Source: Int J Appl Posit Psychol. 2024 Jan 12;9(3):1181–203. doi: 10.1007/s41042-023-00145-y (PMC11519111; doi:10.1007/s41042-023-00145-y)
Supplement: Supplementary file 1 — Supplementary materials (DOCX) [file 41042_2023_145_MOESM1_ESM.docx]

**Supplementary Materials**


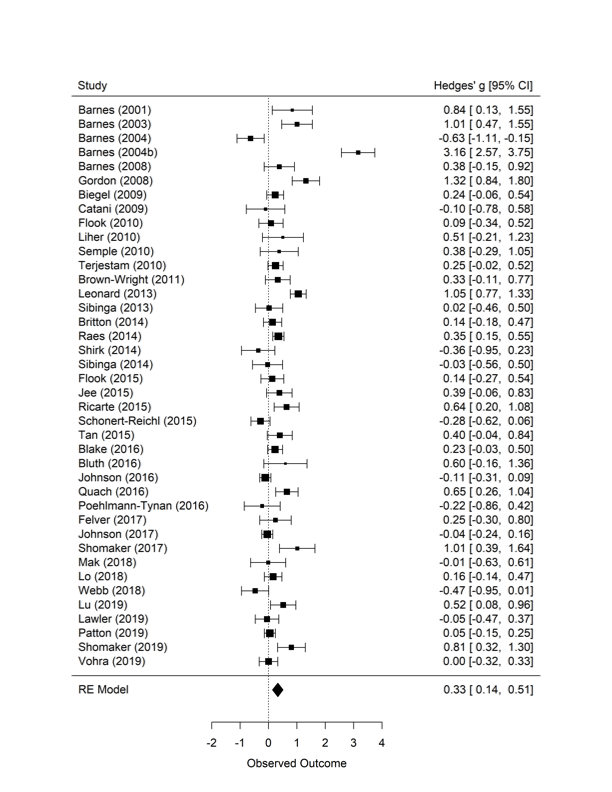


***Figure S1:*** Forest plot depicting individual effect sizes and weighted means

***
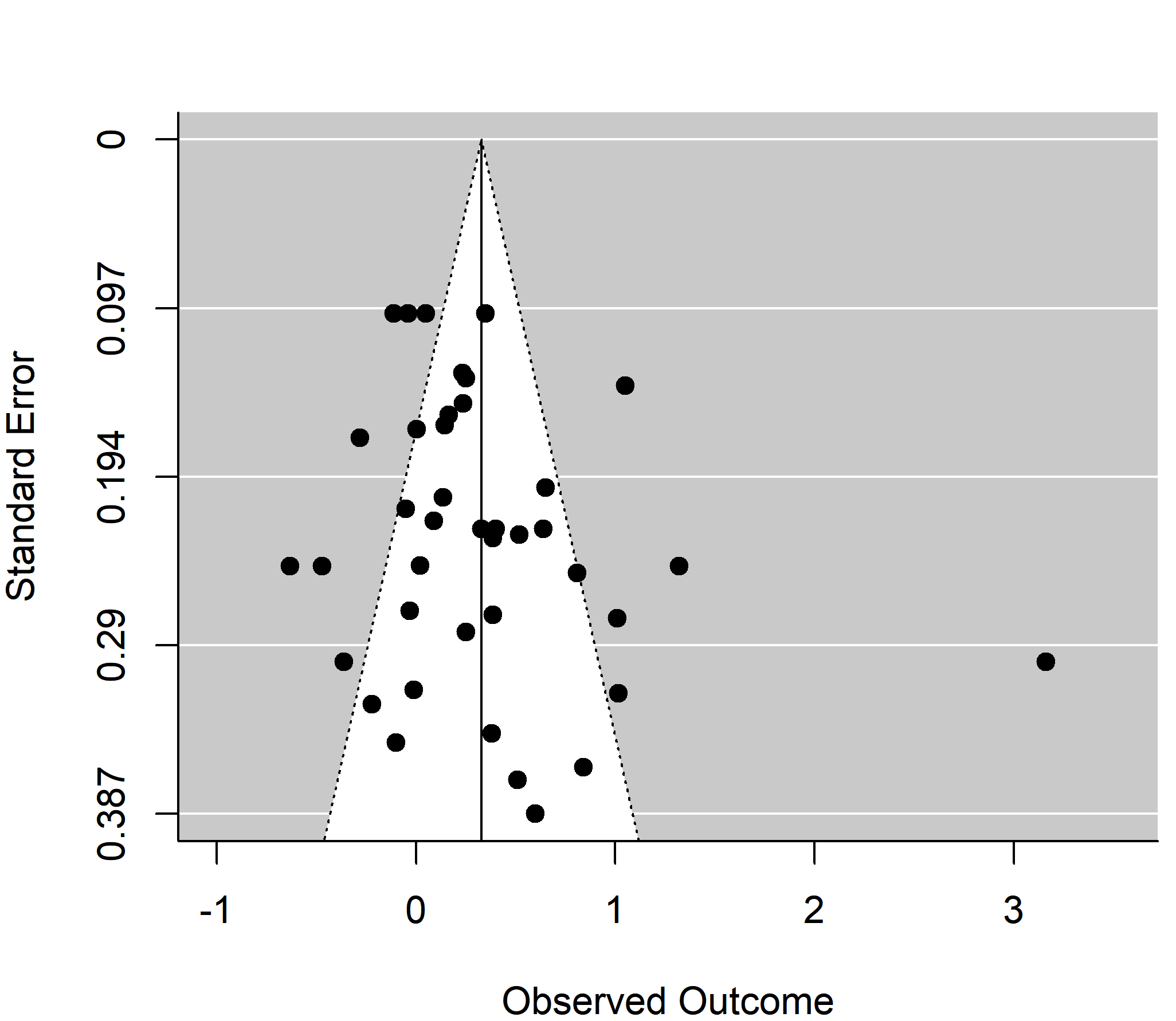
***

***Figure S2:*** Funnel plot representing individual effects against their standard error

| ***Table S1:*** Hypotheses, variables, instruments and risk assessment of studies included in qualitative and quantitative analyses. | | | | | |
| --- | --- | --- | --- | --- | --- |
| First author (year) | Hypotheses/Objectives | Variables | Instruments | Qualitative (Q) and Quantitative (Q’) evidence of risk/protective factors. | Type of risk |
| Barnes (2001) | Reductions in resting blood pressure and total peripheral resistance. Reductions in cardiovascular reactivity to laboratory stressors | 1. Height, weight, waist and hip circumference  2. Blood pressure at rest and in response to a virtual reality car driving simulation task and social stressor interview  3. Cardiac output and heart rate at rest and in response to a virtual reality car driving simulation task and social stressor interview  4. Total peripheral resistance | 1. Stadiometer and scale  2. Dinamap Vital Signs Monitor 1846SX  3. Noninvasive thoracic electrical bioimpedance system (NCCOM-3 Model 6)  4. Calculation from simultaneous blood pressure and total peripheral resistance values | Q:   - Mostly African American youth attending to an inner-city high school - With high normal blood pressure - Excluded self-reported pregnancy - Excluded parental report of congenital heart defect, diabetes, asthma or any chronic illness that requires regular pharmacological intervention   Q’:   - Resting systolic blood pressure in the sitting position ≥85th and ≤95th percentile on three consecutive occasions with respect to age and gender | Socio-economic status  Physical health challenges |
| Barnes (2003) | Reduction in school absenteeism, rule infractions, and days suspended | 1. Experience and expression of anger  2. Days suspended in the previous 4 months  3. Tardy class periods in the previous 4 months  4. Absentee class periods in the previous 4 months  5. Grade percentage  6. Rule infractions  7. Weight/Height/Body mass index  8. TV watched per week. | 1. Spielberger Anger Expression Scale  2.- 6. Teacher-reported  7. Detecto CN220 scale with height rod  8. Self-reported | Q:   - African American youth attending to two inner-city public high schools - With high normal blood pressure - Schools representative of the broader   population of African American high school students in Richmond County  Q’:   - Resting systolic blood pressure ≥85th percentile on 3 consecutive occasions with respect to age, gender and height. | Socio-economic status  Physical health challenges |
| Barnes (2004) | Reductions in resting  and ambulatory blood pressure | 1. Height and weight  2. Resting and ambulatory systolic blood pressure, diastolic blood pressure and heart rate  3. Experience and expression of anger  4. Neighborhood disorder and exposure to violence  5. Physical activity  6. Expectation of benefits | 1. Stadiometer and scale  2. Dinamap 1846SXX monitors and SpaceLabs Model 90207 monitors  3. Spielberger Anger Expression Scale  4. Neighborhood Stress Index  5. Self-reported  6. Likert type scale | Q:   - Seventh-grade middle-school students (African Americans or Caucasians)   Q’:   - No participants were hypertensive (i.e., ≥95th percentile on three pretest evaluation days) based on norms for age, height, and gender - Four participants were ≥95th percentile on one of the (three) evaluation days | Normative risk |
| Barnes (2004b) | Reductions in ambulatory blood pressure | 1. Height and weight  2. Resting and ambulatory systolic blood pressure, diastolic blood pressure and heart rate | 1. Stadiometer and scale.  2. SpaceLabs Model 90207 monitors | Q:   - African American youth attending to five inner-city public high schools - High normal blood pressure   Q’:   - Resting systolic blood pressure ≥85^th^ and ≤95^th^ percentile on 3 consecutive occasions with respect to age, gender and height - Hollingshead Four Factor Social Status Index (M= 34.8-35.7, SD=12.7-13.0). - Single mother head of household (60 -63%) | Socio-economic status  Physical health challenges |
| Napoli (2005) | Increased ability to maintain attention | 1. Attention  2. Social skills  3. General debilitative test anxiety  4. Selective (visual) attention and sustained attention | 1-2. ADD-H Comprehensive Teacher Rating Scale – Attention and Social Skills subscales.  3. Test Anxiety Scale  4. Test of Everyday Attention for Children – Selective attention and sustained attention subtests | Q:  First, second and third grade students at two elementary schools in a U.S. Southwestern city. | Normative risk |
| Barnes (2008) | Reductions in ambulatory blood pressure and overnight urinary sodium excretion. | 1. Height and weight  2. Systolic blood pressure, diastolic blood pressure and heart rate.  3. Overnight urinary sodium excretion rate. | 1. Stadiometer and scale  2. SpaceLabs Model 90207 monitors | Q:   - African American students at risk for essential hypertension attending to two high schools - Parental report of no history of congenital heart defect, diabetes, sickle cell anemia, asthma or any chronic illness or health problem that requires regular pharmacological treatment. - No current or planned engagement in a formal exercise or health promotion program - Having never been pregnant at any point in the study   Q´:   - Resting systolic blood pressure ≥75th and ≤95th percentiles for age, height and sex on three consecutive occasions | Social marginalization  Physical health challenges |
| Gordon (2008) | Reduction in Post Traumatic Stress Disorder symptoms (PTSD) | 1. Trauma-related symptoms  PTSD diagnosis based on 16 trauma symptom questions of the Harvard Trauma Questionnaire. | 1. Harvard Trauma Questionnaire | Q:   - Students attending to high school in a postwar zone in Kosovo   Q’:   - A scoring algorithm based on the Diagnostic and Statistical Manual of Mental Disorders-Fourth Edition (DSM-IV) criteria that uses Harvard Trauma Questionnaire scores to define PTSD presence | War  Mental health challenges |
| Biegel (2009) | Reduction in stress-related symptoms (depression, anxiety and sleep difficulties), improvement in self-esteem, diagnosis and global psychological functioning | 1. DSM-IV-TR Psychiatric diagnoses  2. Level of general psychological and social functioning  3. Diagnostic change variable (number of conditions diagnosed at pretest and increase or decrease in number of diagnoses)  4. Number of individual and group psychotherapy visits  5. Number of mental health hospitalizations  6. Type of mental health medication and medication dosage  7. Perceived stress  8. State/Trait anxiety  9. Psychological symptoms: somatization, depression, anxiety, hostility, obsessive-compulsive, interpersonal sensitivity  10. Self-Esteem.  11. Reasons for ending treatment  12. Alcohol and illicit drug use  12. Program evaluation | 1.- 3 DSM-IV-TR  4. - 6. Clinician-reported  7. Perceived Stress Scale (PSS-10)  8. State/Trait Anxiety Inventory (STAI-C)  9. Hopkins Symptom Checklist 90 Revised (SCL-90-R)  10. Rosenberg Self-Esteem Scale (SES)  11. Reasons for Ending Treatment Questionnaire  12. Self-report | Q:   - Adolescents with current or past psychiatric care (without time restrictions) - Excluded current substance abuse (drug or alcohol) or past or present psychiatric or neurological disorders that would preclude or severely limit participation in the study   Q’:   - DSM-IV psychiatric diagnoses (Axis I: Clinical Disorders) and current level of general psychological and social functioning (Axis V: Global Assessment of Functioning) obtained from individual clinicians’ reports | Mental health challenges |
| Catani (2009) | Reduction in PTSD symptoms, functioning problems and physical health symptoms | 1. PTSD severity  2. 3-weeks after tsunami PTSD provisional diagnosis  3. Functional impairment in different areas of children’s life  4. Severity of tsunami exposure.  5. Presence of somatic complaints in last 4 weeks | 1. - 2. UCLA PTSD Index for DSM-IV in interview form  3. 5 questions on functioning (number of “yes”)  4. 5 questions on exposure (number of “yes”)  5. 5 questions on somatic complaints (number of “yes”) | Q:   - Living at two provisional refugee camps three weeks after a tsunami. - Region severely affected by the Sri Lanka civil war. - No mental retardation, psychosis or neurological disorder   Q’:   - UCLA PTSD Index for DSM-IV score used for provisional PTSD diagnosis - 16.12 % lost father, 69.67% lost mother and 32.25 % affected by traumatic war experiences | Natural disaster  War  Mental health challenges |
| Barnes (2010) | Reductions in resting and daytime blood pressure and heart rate | 1. Resting and daytime blood pressure and heart rate  2. Cohesion, emotional expressiveness and conflict  3. Expectation of health benefits | 1. Dinamap Vital Signs Monitor 1846SX  2. Family Relations Index from the Family Environment Scale  3. Three ad hoc questions | Q:   - African American students at risk for essential hypertension - Report of no history of congenital heart defect, diabetes, sickle cell anemia, asthma or any chronic illness or health problem that requires regular pharmacological treatment. - No current or planned engagement in a formal exercise or health promotion program. - No pregnant   Q´:   - Resting systolic blood pressure ≥ 50th and ≤ 95th percentiles for age, height and sex on three consecutive occasions - Socioeconomic status based on Hollingshead four factor indexes (M = 33.47; SD = 11.96) | Social marginalization |
| Flook (2010) | Improvement in executive functions (greater improvement for children with poorer executive functioning) | 1. Executive functioning | 1.Behavior Rating Inventory of Executive Function - Metacognition Index, Behavioral Regulation Index and Global Executive Composite | Q:   - Second- and third- grade children at an on-campus university elementary school in Los Angeles | Normative risk |
| Liehr (2010) | Reduction in depressive and anxiety symptoms | 1. Depressive symptoms  2. Anxiety symptoms | 1. Short Mood and Feelings Questionnaire  2. State Anxiety Inventory for Children | Q:   - Minority children from a summer camp   Q’:   - 64% were from Caribbean and Central American countries | Socio-economic status  Social marginalization |
| Mendelson (2010) | Reduction in involuntary stress responses and improvement in mood and relationships with peers and teachers. | 1. Involuntary stress responses  2. Depressive symptoms  3. Positive and negative emotions  4. Relations with peers and school. | 1. Responses to Stress Questionnaire  2. Short Mood and Feelings Questionnaire – Child Version  3. Emotion Profile Inventory  4. People in My Life - Friends and School factors scales | Q:   - Fourth and fifth grade students recruited from four Baltimore City public elementary schools (inner-city youth)   Q’: Depressive symptoms | Socio-economic status  Mental health challenges |
| Semple (2010) | Reduction in attention problems, anxiety symptoms and behavior problems | 1. Attention problems  Behavior problems  Internalizing problems  2. Anxiety symptoms | 1. Child Behavior Checklist: Parent Report Form (CBCL) – CBCL Attention problems subscale, CBCL Total Problems Scale, CBCL internalizing problems scale  2. Multidimensional Anxiety Scale for Children–Total anxiety score.  STAI-C state anxiety scale | Q:   - English-speaking inner-city children enrolled in a university clinic-based remedial reading tutoring program referred by the clinic’s educational psychologist as having significant reading difficulties - Most displayed some indicators of associated stress or anxiety | Physical health challenges |
| Terjestam (2010) | Improvement in well-being at school and self-image and reduction in psychologic distress and general stress | 1. Well-being at school  2. Psychological distress  3. Self-image (physical characteristics; skills and talents; psychologic well-being; relation to parents; relation to others; total score)  4. General stress: at home, at school and among friends. | 1. Well-being at school  2. Psychologic distress scale  3. Test “I think that I am”  4. Three statements to assess general stress | Q:   - Sixth grade students attending to school | Normative risk |
| Brown-Wright (2011)^1^ | Reductions in ambulatory blood pressure and self-reported hostility | 1. Systolic blood pressure, diastolic blood pressure and heart rate  2. Hostility | 1. Dinamap 1846SXX monitors and SpaceLabs Model 90207 monitors  2. Cook-Medley Hostility Inventory- Adolescent version | Q:   - African American students at risk for essential hypertension - Parental report of no history of congenital heart defect, diabetes, sickle cell anemia, asthma or any chronic illness or health problem that requires regular pharmacological treatment - No current or planned engagement in a formal exercise or health promotion program - Having never been pregnant at any point in the study   Q´:   - 39% married parents - Resting systolic blood pressure between the 50th and 95th percentiles for age, height and sex on three consecutive occasions - Socioeconomic status based on Hollingshead four factor indexes (M = 33.47; SD = 11.96) | Social marginalization |
| Gregoski (2011)^1^ | Reductions in ambulatory blood pressure and overnight urinary sodium excretion | 1. Systolic blood pressure, diastolic blood pressure and heart rate  2. Overnight urinary sodium excretion rate  3. Perceived stress | 1. Spacelab 90207 monitors  3. PSS | Same as Brown-Wright (2011) | Social marginalization |
| White (2012) | Reductions in perceived stress and improvements in coping, self-esteem and self-regulation | 1. Perceived stress  2. Coping  3. Self-esteem  4. Mindfulness – Healthy self-regulation | 1. Feel Bad Scale  2. Schoolagers´s Coping Strategies Inventory  3. Self-Perception Profile for Children - Global Self-Worth subscale  4. Mindful Thinking and Action Scale for Adolescents – Healthy Self Regulation subscale | Q:   - Fourth- and fifth-grade girls attending public schools. - Able to pay attention for 1 hour, able to participate in physical poses - No developmental disorder as determined by the need for special education one-to-one assistance   Q’:   - Most families reported no recent family stress (85.1%) and no current participant health problem (85.1%). Reported stressors: grandparent illness, family death, recent move, unemployed parent, or resolving domestic abuse. Reported health problems: asthma, allergies, headaches, back pain, and history of broken bones | Normative risk |
| Evans-Chase (2013) ^2^ | Improvements in self-regulation | 1. Self-regulation – interpersonal restraint  Self-regulation – intrapersonal restraint | 1. Restraint-Weinberger Adjustment Inventory – Composite score, Suppression of Aggression subscale and Impulse Control subscale. | Q:   - Youth incarcerated in a long-term juvenile justice facility | Social marginalization  Out of home placement |
| Leonard (2013) | Improvement in attentional capabilities | 1. Offending history.  2. Attention regulation: conflict monitoring, orienting and alerting, overall task performance (response time, and accuracy) and intra-individual response variability | 1. Self-report of Offending  2. Attention Network Task | Q:   - Incarcerated male youth in a high-stress, high-security correctional facility   Q’:   - 98% were Black or Latino - Self-report of offending behavior indicated that 74% reported ever engaging in non-violent offenses (e.g., theft, selling drugs) and 54% reported engaging in violent offenses (e.g. murder, assault) | Out of home placement |
| Mano (2013) | Reductions in pain, youth- and parent- reported anxiety, functional disability and pain catastrophizing, improvements in health-related quality of life | 1. Frequency, severity and duration of pain episodes 2. State and trait anxiety 3. Levels of self-efficacy 4. Health-related quality of life (physical, emotional, social and school) 5. Thoughts and feelings when in pain 6. Functional disability   7. Expectations about the benefits of treatments | 1. Pain Frequency-Severity Duration scale  2. STAI-C  3. Mindfulness Self-Efficacy Scale  4. Quality of Life Inventory  5. Pain Catastrophizing Scale for Children  6. Child Activity Limitations Questionnaire  7. Five-item rating scale | Q:   - Adolescents attending to an outpatient, multidisciplinary, pain clinic currently being treated for a pediatric, chronic pain condition of a 3-month or greater duration - Excluded diagnosed with cognitive disability or presenting current suicidality or serious mental illness   Q´:   - Pain frequency: Control (M = 6.50 days/two weeks) MBT (everyday). Pain severity: Control (6/10, SD=1.41) MBT (5.33/10, SD=2.02). Worst pain duration: Control (1-2 hours/day) MBT (9-12 hours/day) | Physical health challenges |
| Sibinga (2013) | Reductions in psychological symptoms and enhanced coping. | 1. Psychological functioning.  2. Coping.  3. Anxiety.  4. Standard sleep parameters: latency to sleep onset; awakenings; time awake after sleep onset (WASO); total sleep time; sleep efficiency.  5. Total daily salivary cortisol at awakening, 60 min post-awakening; 2:30 pm and bedtime.  6. Mindfulness. | 1. SCL-90-R.  2. COPE inventory  3. Multidimensional Anxiety Scale for Children  4. Paper sleep diaries, Respironics, Mini Mitter Actiwatch  5. Salivette device  6. Not reported | Q:   - Seventh and eighth grade boys attending tuition-free middle school for urban boys with financial need and academic potential. - Not in foster care. - Not having significant psychopathology, developmental delay, substance abuse or behavioral problems identified by school staff. | Socio-economic status |
| Britton (2014) | Reductions in internalizing, externalizing and attention problems and self-injurious behavior and suicidal ideation. Improvements in mindfulness. | 1. Internalizing problems.  2. Externalizing problems.  3. Attention problems.  4. Frequency of self-injurious behavior and suicidal ideation.  5. Total Affect Disturbance.  6. Positive affect.  7. Mindfulness.  8. Acceptability of the meditation practices | 1. – 4. Youth Self Report (YSR) - Internalizing Problems subscale, Externalizing Problems subscale, Attention Problems subscale  5. STAI-C  6. STAI-C positive items alone.  7. Cognitive and Affective Mindfulness Scale  8. Journals. | Q:   - Sixth-grade students attending school. | Normative risk |
| Parker (2014) | Improvements in cognitive, emotional and behavioral regulatory abilities and reductions in intentions to use substances. | 1. Executive function.  2. Intentions to use alcohol and tobacco.  3. Adaptive functioning and behavioral/emotional problems in the classroom. | 1. Flanker Fish task.  2. Intentions to Use Alcohol and Tobacco scale.  3. Children’s Behavior checklist-Teachers Report Form. | Q:   - Children attending two elementary schools. | Normative risk |
| Raes (2014) | Reductions in depressive symptoms. | 1. Depression symptoms. | 1. Depression Anxiety Stress Scales (DASS-21) | Q:   - Students attending to years 3 to 6 in Flemish secondary schools.   Q’:   - 88 participants with DASS-21-D scores of moderate depression or greater at pretest: 41 (21%) MBT, 47 (24%) Control. | Mental health challenges |
| Shirk (2014) | Reductions in depressive symptoms. | 1. DSM-IV Axis I disorders: major depressive disorder, dysthymic disorder, bipolar disorder, posttraumatic stress disorder and substance dependence.  2. Trauma.  3. Depression symptoms.  4. Disruptive behavior problems.  5. Verbal Intelligence.  6. Therapist interventions.  7. Therapist adherence to the m-CBT protocol.  8. Client satisfaction with clinical services.  9. Treatment acceptability. | 1. Kiddie-Schedule for Affective Disorders and Schizophrenia-Present and Lifetime Version.  2. Trauma Experiences Screening Inventory-Child version  3. Beck Depression Inventory – Second Edition  4. CBCL  5. Wechsler Adult Intelligence Scale-IV or Wechsler Intelligence Scale for children-IV. Similarities subtest and Block Design subtest  6. Therapy Process Observational coding System – Strategies Scale.  7. Adherence Checklist  8. Client Satisfaction Questionnaire  9. Treatment Evaluation Inventory. | Q:   - Adolescents referred for outpatient treatment at a large urban community mental health center with two clinics. - Met diagnostic criteria for a depressive disorder, dysthymic disorder, or depressive disorder-not otherwise specified based on structured diagnostic interview. - No attempted suicide or engaged in severe self-injurious behavior within the past 3 months, no met diagnostic criteria for bipolar disorder and/or substance dependence disorder, no presented psychotic symptoms.   Q’:   - 49% non-Hispanic Caucasian, 33% Hispanic, 38% African American. - All reported at least one incident of physical abuse (49%), witnessing family violence (58%), sexual abuse (67%) and verbal/emotional abuse (47%) in response to a highly structured screening interview. - A majority of the sample reported more than one type of interpersonal trauma throughout their lifetime: one type (23%), two types (28%), three or more types (49%). - No intellectual deficit (IQ<70). - 46% met full diagnostic criteria for posttraumatic stress disorder. - 14% reported using illegal substances at least 3 times a week. | Mental health challenges  Interpersonal trauma. |
| Sibinga (2014) | Reductions in psychological difficulties (e.g., depression and hostility) and stress and improvements in coping. | 1. Psychological symptoms: General severity, Somatization, Depression, Anxiety, Hostility, Paranoid ideation, Obsessive-compulsive, Interpersonal Sensitivity, Phobic anxiety, Psychoticism.  2. Positive and Negative affect.  3. State trait anxiety.  4. Health and quality of life: Satisfaction, Comfort, Risk Avoidance, Resilience and Achievement.  5. Perceived stress.  6. Coping styles: problem-focused coping, wishful thinking, detachment, seeking social support, focusing on the positive, self-blame, tension reduction and keeping to self.  7. Coping self-efficacy.  8. General self-Efficacy.  9. Self esteem. | 1. SCL-90-R.  2. Positive and Negative Affect Schedule  3. STAI-C.  4. Child Health and Illness Profile Adolescent Edition  5. PSS.  6. Ways of Coping Scale  7. Coping Self-Efficacy Scale  8. General Self-Efficacy Scale  9. Self-esteem scale. | Q:   - Urban youth attending to an outpatient primary care clinic of a large, urban tertiary care academic hospital - Not significant cognitive, behavioral and psychiatric disorders or substance abuse assessed by their health care provider. - 44 % families received food stamps.   Q’:   - Over 90% African American patients, approximately half of parents unemployed and over 85% enrolled in Medicaid. | Socio-economic status |
| van de Weijer-Bergsma (2014) | Reductions in stress, improvements in well-being and mental health. | 1. Ruminative and repetitive thoughts.  2. Emotional functioning: differentiating emotions, verbal sharing of emotions, not hiding emotions, bodily awareness, attending to others’ emotions, and analyses of emotions.  3. Sense of coherence.  4. Happiness.  5. Anxiety symptoms.  6. Anger/aggression, social competence, and anxiety/withdrawal.  7. Disorders of initiating and maintaining sleep, disorders of arousal nightmares, sleep–wake transition disorders, and disorders of excessive somnolence.  8. Class Climate. | 1. Non-Productive Thoughts Questionnaire for Children.  2. Emotion Awareness Questionnaire.  3. Sense of Coherence Questionnaire for Children  4. Subjective Happiness Scale  5. Screen for Child Anxiety Related Emotional Disorders– parent report  6. Social Competence and  Behavior Evaluation– parent report  7. Sleep Disturbance Scale for Children – parent report  8. School as a Caring Community Profile II – teacher report | Q:   - Students attending to three public elementary schools in Amsterdam.   Q’:   - 10%, 30% and 81% of the students coming from immigrant families, respectively. | Normative risk |
| Atkinson (2015) | Reduced eating disorder symptoms. | 1. Body mass index.  2. Weight and shape concern.  3. Negative affect.  4. Dietary restraint.  5. Eating disorder symptoms.  6. Psychosocial impairment.  7. Psychosocial Impairment.  8. Mindful acceptance and awareness. | 1. Self-reported height and weight.  2. Eating Disorder Examination Questionnaire (EDE-Q).  3. Positive and Negative Affect Schedule-Expanded  4. Dutch Eating Behaviour Questionnaire-Restraint  5. Socio-cultural Attitudes Towards Appearance Scale Internalization – General and Pressures subscales.  6. Nine items from the EDE-Q.  7. Clinical Impairment Assessment  8. Child and Adolescent Mindfulness Measure (CAMM). | Q:   - Female adolescents attending to all single-sex girls’ high schools. | Normative risk |
| Evans-Chase (2015) ^2^ | Improvement in mindfulness. | 1. Facets of Mindfulness: Observing, Describing, Acting with Awareness, Nonjudging of Inner Experience and Nonreactivity to Inner Experience. | 1. The 5-Facet mindfulness Questionnaire – Total score and Total score without Observe subscale. | Q:   - Male juvenile offenders incarcerated in the New Jersey state correctional facility who were scheduled for release no less than 3 months from entrance into the study. - Excluded residents in protective custody or not safe in a group setting (for being a threat to others or a high victim risk defined by the facility). | Social marginalization  Out of home placement |
| Flook (2015) | Improvements in cognitive and behavioral outcomes. | 1. Social competence: overall, prosocial behavior, emotion regulation.  2. Sharing  3. Delay of gratification  4. Cognitive flexibility  5. Inhibitory control  6. Grades – learning, health, social/emotional, cognition, language | 1. Teacher Social Competence Scale: Total scale, Prosocial Behavior subscale, Emotion Regulation subscale.  2. Sharing task  3. Delay of gratification task  4. Dimensional change card sort task  5. Flanker task  6. School grades | Q:   - Six different elementary schools within a public school district in a medium-sized Midwestern city.   Q’:   - 37.9% of children are considered socioeconomically disadvantaged. | Normative risk |
| Himelstein (2015) | Improvements in mindfulness, locus of control, decision-making, self-esteem, perceived risk of drug use and behavioral regulation. | 1. Mindfulness.  2. Locus of Control.  3. Decision-making.  4. Self-Esteem.  5. Perceived risk of drug use.  6. Behavioral Regulation. | 1. Mindfulness Attention Awareness Scale (MAAS).  2. Prison Locus of Control Scale  3. Decision-making Skills  4. SES.  5. Monitoring the Future Questionnaire.  6. Third-person observations and ratings. | Q:   - Incarcerated adolescents in a juvenile detention camp, charged with both violent and non-violent offenses and met mental health diagnostic criteria for a number of diagnoses including posttraumatic stress disorder and other anxiety disorder, attention deficit hyperactive disorder, learning disorders, adjustment disorders and mood disorders. - All participants met criteria for a substance use disorder.   Q’:   - 70% Latino, 14% African American, 6% Caucasian, 5% Pacific Islander and 5% of mixed-ethnic descent. | Social marginalization  Out of home placement  Mental health challenges |
| Jee (2015) | Reductions in stress using psychological and physiological techniques. | 1. Overall score of clinical significance, attention, externalizing problems and internalizing problems.  2. Acceptance and Mindfulness.  3. Changes in stress.  4. Heart rate variability indices from the time and frequency domains. | 1. Pediatric Symptom Checklist 17  2. CAMM  3. STAI-C.  4. Electroencephalogram recording. | Q:   - Being in foster care or kinship care attending to a pediatric medical home (clinic).   Q’:   - PCS-17 scores: much lower levels of stress than national assessment reports for foster youth at baseline and post-intervention. | Out of home placement |
| Ricarte (2015) | Reductions in anxiety and improvements in attention and concentration. | 1. State/Trait anxiety.  2. Sustained attention and eye-hand coordination speed (Part A). Mental flexibility and ability to switch between two sets of cognitive stimuli (Part B).  3. Focused attention.  4. Memory span, concentration and sustained attention. | 1. STAI-C.  2. Trail Making Test  3. Perception of Differences Test-Faces (Faces-R).  4. Wechsler Intelligence Scale for children III – Digit Span Test. | Q:   - Children from a rural area attending to school. - The socioeconomic and cultural profile of the area is low. - The population is dominated by illiterate older adults over the age of 75. - Economic activity focuses on the primary sector (mainly agriculture). - Household income is low in comparison with the national average.   Q’:   - A total of 98% of the younger population only completed formal education to primary level. | Normative risk |
| Schonert-Reichl (2015) | Improvement in executive functions, stress regulation, social-emotional competence and school achievement. | 1. Executive functions (accuracy and reaction time on two tasks)  2. Salivary cortisol  3. Empathy concern and perspective-taking  4. Optimism  5. Emotional control  6. School self-concept  7. Depressive symptoms.  8. Mindfulness  9. Social responsibility.  10. Pro-sociality.  11. Peer acceptance.  12. Math achievement. | 1. Flanker task  1. Hearts and flowers task  2. Dental cotton rolls  3. Interpersonal Reactivity Index  4 - 5. Resiliency Inventory - Optimism subscale and Emotional control subscale  6. Marsh´s Self-Description Questionnaire - School self-concept subscale  7. Seattle Personality Questionnaire for Children - Depressive symptoms subscale  8. MAAS  9. Social responsibility subscale of the Social Goals Questionnaire.  10. Peer nominations of pro-sociality.  11. Peer nominations of peer acceptance.  12. Math grades from school records. | Q:   - Fourth and fifth grade children attending to elementary school in a suburban, predominantly middle-class community near a large western Canadian city.   Q’:   - The average income for the neighborhoods in which each of the four schools was located approximated the median annual income for Canada ($52,800; Statistics Canada, 2006). Regarding children’s family composition, 84% reported living in two-parent homes (including both biological and step-parent families), 9% reported living with mother only, and the remainder reported living in dual-custody arrangements (i.e., half time with mother, half time with father). - With regard to language, 66% of the children reported that English was their native language. For the remaining children, the majority reported that their language at home was of East Asian origin (25%; e.g., Korean, Mandarin, Cantonese), and the remaining 10% indicated a range of other languages (e.g., Spanish, Russian, Polish). | Normative risk |
| Tan (2015) | Reductions in stress, depression, anxiety and cognitive inflexibility and improvements in self-esteem and resiliency. | 1. Depression symptoms.  2. Self-Esteem.  3. Resiliency.  4. Mindfulness.  5. Psychological inflexibility.  6. Parent/carer’s report of adolescent’s behavioral problems. | 1. DASS-21  2. SES.  3. Resiliency Scales for Children and Adolescents  4. CAMM  5. Avoidance and Fusion Questionnaire for Youth  6. CBCL Total. | Q:   - Adolescents from three public community child and adolescent mental health clinics in a major east-coast city of Australia. - No intellectual impairment, organic brain syndrome, chronic substance abuse, acute suicidality and psychosis.   Q’:   - An Axis I mental health diagnoses identified by the International Classification of Diseases, Tenth edition. | Mental health challenges |
| Blake (2016) ^3^ | Reductions in anxious and depressive symptoms and improvements in subjective and objective indices of sleep. | 1. Six domains of anxiety: generalized anxiety, panic/agoraphobia, social phobia, separation anxiety, obsessive-compulsive disorder, physical injury fears, and total score.  2. Depressive symptoms.  3. DSM-IV Axis I disorders: depression, mania, psychosis, panic disorder, social phobia, specific phobia/agoraphobia, generalized anxiety, obsessive-compulsive disorder, separation anxiety and posttraumatic stress disorder.  4. Subjective sleep quality and disturbances and impact of poor sleep on functioning: Total sleep time, Sleep onset latency, Sleep efficiency, Global PSQI  5. Daytime sleepiness.  6. Objective measures (average scores and variability scores, across the week): Total sleep time (TST), Sleep onset latency (SOL), Sleep efficiency (SE), Wake after sleep onset (WASO), Bedtime (BT) | 1. Spence Children’s Anxiety Scale (SCAS).  2. Center for Epidemiologic Studies – Depression Scale  3. Kiddie Schedule of Affective Disorders and Schizophrenia Children’s Version – Present and Lifetime Version  4. The Pittsburgh Sleep Quality Index (PSQI)  5. Pediatric Daytime Sleepiness Scale  6. Actiwatch  6. Paper sleep diary | Q:   - Students attending to secondary schools in the Melbourne metropolitan Area, with high levels of anxiety and sleeping difficulties. - Had never met criteria for Major Depressive Disorder - No current or past diagnoses of bipolar or psychotic disorder, history of head injury, current use of anxiolytic or hypnotic medications, inadequate comprehension of written and spoken English.   Q’:   - Ratings on questionnaire indicating high anxiety (SCAS Total Score >32 males and > 38 females) as well as likely presence of sleep problems (PSQI Global Score > 4). | Mental health challenges |
| Bluth (2016) | Improvements in psychosocial well-being. | 1. Mindfulness  2. Self-compassion  3. Social connectedness  4. Perceived stress  5. Anxiety  6. Depression | 1. CAMM  2. Self-Compassion Scale-Short Form  3. Social Connectedness Scale.  4. PSS.  5. STAI-C.  6. Short Mood and Feelings Questionnaire. | Q:   - High-risk adolescents who have struggled academically within the traditional public high school system and were referred to an alternative high school.   Q´:   - School racially/ethnically diverse (54% Hispanic, 24% African-American, 18% Caucasian, 3% other) and primary low income (88% received a free or reduced lunch). 40% court involved, 67% had prior suspensions, 24% with mental health support, 87% failing >= class in original school, 18% parenting a child or pregnant. | Socio-economic status |
| Daly (2016) | Reductions in BMI. | 1. Weight and BMI.  2. Mindful awareness.  3. Motivation for participation. | 1. Scale  2. MAAS  3. Adolescent Motivation Questionnaire | Q:   - Females attending to an urban public high school.   Q’:   - BMI>90th percentile (overweight/obese). | Physical health challenges |
| Johnson (2016) | Reductions in anxiety, depression, weight/shape concerns and improvements in wellbeing. | 1. Negative affect.  2. Weight and shape concern.  3. Well-being.  4. Mindfulness.  5. Emotional dysregulation.  6. Self-compassion. | 1. DASS-21  2. EDE-Q  3. Warwick-Edinburgh Mental Wellbeing Scale  4. CAMM  5. Emotional Regulation Scale (DERS).  6. Self-compassion scale | Q:   - Adolescents attending to urban coeducational secondary and primary schools.   Q’:   - Participating schools represented a broad range of socioeconomic demographics measures on the Index of Community Socio-Educational Advantage, ranging from 951 to 1160 (M= 1047, SD = 85.77). Two low SES schools (within one SD below the mean), two medium SES schools (within one SD above the mean), one high SES school (greater than one SD above the mean). 16.2% low SES students, 39% medium SES students, 44.8 high SES students. 21.6% scored in the clinical range for depression, 22.2% for anxiety. | Normative risk |
| Poehlmann-Tynan (2016) | Improvements in empathic and compassionate responding, self-regulation and executive functions. | 1. Empathic responses to simulated stress.  2. Empathic and compassionate responding within the family context.  3. Integrated self-regulation skills.  4. Attentional focus, impulsivity and inhibitory control. | 1. Distress Task.  2. Attachment Story Completion Task  3. Head-Toes-Knees-Shoulders Task  4. Go/No-Go Task | Q:   - Children from five classrooms across three low-income, federally subsidized full-day preschools in a mid-sized Midwestern city. - They were already participating in a dialogic reading program designed to support school readiness in children from low-income families (parents had to report their income upon registering at the school to determine eligibility).   Q´:   - Seventy-two percent (n=21) of the study children were non-White and 100 % were living in poverty, according to federal guidelines. | Socio-economic status |
| Quach (2016) | Improvements in working memory capacity and mindfulness and reductions in perceived stress and anxiety. | 1. Working memory capacity.  2. Perceived Stress.  3. Anxiety symptoms.  4. Mindfulness | 1. Automated Operation Span Task  2. PSS-10.  3. Screen for Child Anxiety and Related Emotional Disorders  4. CAMM | Q:   - Adolescents attending to a large public junior high school. - Students were predominately from low-income minority households. - No hearing impairments, injuries or physical disabilities that would hinder full participation in either intervention.   Q’:   - About 80% eligible for a free or reduced lunch. | Socio-economic status |
| Sibinga (2016) | Improvements in psychological functioning. | 1. Mindfulness.  2. Stress and positive/coping  3. Depressive symptoms  4. Psychological symptoms (paranoid ideation, hostility, somatization)  5. Anxiety  6. Positive and negative affect  7. Differential emotions (interest, enjoyment, sadness, anger, guilt, contempt, fear , self-hostility, shame shyness)  8. Aggression.  9. Anger expressivity (temperamental expressivity, reactive expressivity).  10. Response style/coping (rumination, distraction, problem solving)  11. Coping (positive and negative coping).  12. Coping self-efficacy.  13. Posttraumatic symptoms. | 1. Children’s Acceptance and Mindfulness Measure  2. Perceived Stress Scale  3. Children’s Depression Inventory-Short Form  4. SCL-90-R.  5. Multidimensional Anxiety Scale for Children  6. Positive and Negative  Affect Schedule  7. Differential Emotions Scale  8. Aggression scale  9. State-Trait Anger Expression Inventory  10. Response Style Questionnaire  11. Brief COPE  12. Coping Self-Efficacy  Scale  13. by using the Children’s  Post-Traumatic Symptom Severity  Checklist | Q:   - Seventh and eighth grade students attending to two Elev8 Baltimore schools.   Q’:   - Approximately 99% of participants were eligible for free or reduced meals | Normative risk  Socio-economic status |
| Blake (2017a) ^3^ | Reductions in symptoms of anxiety and improvements in subjective and objective indices of sleep, sleep hygiene awareness and presleep hyperarousal on school nights. | 1. Objective measures (average scores and variability scores, on school nights): total sleep time, sleep onset latency, sleep efficiency, wake after sleep onset, bedtime  2. Subjective measures: Bed time, sleep onset time, number of nocturnal awakenings, wake time and rise time.  3. Subjective sleep quality and disturbances, impact of poor sleep on functioning  4. Anxiety symptoms  5. Somatic and cognitive arousal before sleep  6. Sleep hygiene awareness  7. Past or present psychopathology | 1. Actiwatch  2. Paper sleep diary  3. PSQI  4. SCAS  5. Pre-Sleep Arousal Scale (PSAS)  6. Sleep beliefs scale  7. The Kiddie Schedule of Affective Disorder and Schizophrenia Children’s Version – Present and Lifetime Version | Same as Blake (20016) | Mental health challenges |
| Blake (2017b)^3^ | Improvements in social problems, attention problems and aggressive behaviors | 1. Social problems  2. Attention problems  3. Aggressive behaviors | 1. CBCL Social Problems, Attention problems, Aggressive Behavior. | Same as Blake (20016) | Mental health challenges |
| Felver (2017) | Improvements in attention regulation. | 1. Attention regulation: conflict monitoring, orienting and alerting. | 1. Attention Network Task | Q:   - Families self-enlisted in a database or recruited via flyers. No reported psychological diagnosis. No reported epilepsy or seizures.   Q’:   - 98% were reportedly of European American ethnicity. Yearly household income from US$9,000 to US$250,000, median of US$46,500. 77.1% two-parent households. | Normative risk |
| Johnson (2017) | Reductions in anxiety, depression, weight/shape concerns and improvements in wellbeing and mindfulness | 1. Negative affect  2. Weight and shape concern  3. Well-being  4. Mindfulness | 1. DASS-21  2. EDE-Q  3. Warwick-Edinburgh Mental Wellbeing Scale  4. Comprehensive Inventory of Mindfulness Experiences-Adolescents | Q:   - Adolescents attending to four urban coeducational secondary schools.   Q’:   - Participating schools represented a broad range of socioeconomic demographics measures on the Index of Community Socio-Educational Advantage, ranging from 959 to 1144 (M= 1061.50, SD = 76.41). | Normative risk |
| Shomaker (2017)  Shomaker (2019a) | Reductions in depressive symptoms and insulin resistance | 1. Mindfulness  2. Depressive symptoms  3. Major psychiatric disorders  4. Trait anxiety  5. Perceived Stress  6. Serum insulin concentrations  7. Serum glucose concentrations  8. Height and weight  9. Breast development | 1. MAAS  2. The Center for Epidemiologic Studies – Depression Scale  3. The Kiddie Schedule of Affective Disorder and Schizophrenia Children’s Version – Present and Lifetime Version  4. STAIC – Trait Version  5. PSS  6. Radioimmunoassay  7. 2300 STAT Plus Glucose Lactate Analyser  8. Stadiometer and scale  9. Self-reported Tanner stage | Q:   - Female adolescents, parent-reported T2D, prediabetes, or gestational diabetes in at least 1 first- or second-degree relative, good general health. - No major psychiatric disorder, no major medical problem, no medication use affecting insulin resistance or mood, no structured weigh loss or psychotherapy and no pregnancy   Q’:   - Female adolescents with over-weight/obesity (BMI>= 85^th^ percentile), mild-to-moderate depressive symptoms (>=16 on the 20-item Center for Epidemiologic Studies-Depression scale). No T2D (fasting glucose level > 126 mg/dL). | Physical health challenges  Mental health challenges |
| Blake (2018) ^3^ | Improvements in objective and self-reported indices of sleep | 1. Objective measures: total sleep time, sleep onset latency, sleep efficiency, wake after sleep onset, bedtime  2. Subjective measures: bed time, sleep onset time, number of nocturnal awakenings, wake time and rise time  3. Subjective sleep quality and disturbances and impact of poor sleep on functioning  4. Anxiety symptoms  5. Depressive symptoms  6. Past or present psychopathology  7. Self-efficacy | 1. Actiwatch  2. Paper sleep diary  3. PSQI  4. SCAS  5. The Center for Epidemiologic Studies – Depression Scale  6. The Kiddie Schedule of Affective Disorder and Schizophrenia Children’s Version – Present and Lifetime Version  7. General self-Efficacy Scale | Same as Blake (2016) | Mental health challenges |
| Mak (2018) | Improvements in attention and other attention variables, executive function, physical and psychological outcomes | 1. Sustained attention  2. Inattention  3. Vigilance  4. Selective attention  5. Working memory  6. Attentional switching and inhibition.  7. Selective attention and working memory  8. Day-to-day executive functioning  9. Mindfulness  10. Behaviour and psychological attributes  11. Lower limb functional strength  12. Flexibility  13. Submaximal motor capacity  14. Mobility limitations  15. Quality of life  16. Pain experience | 1-5. Conners’ Continuous Performance Test (CCPT)  6. Delis-Kaplan Executive Function System - Colour-word interference test and trail making test  7. Wechsler Intelligence Scalefor Children – Digit span and symbol search subtests.  8. Behaviour Rating Inventory of Executive Function  9. Child and Adolescent Mindfulness Measure  10. Strengths and Difficulties Questionnaire  11. Sit-to-stand test, lateral step-up test and half-kneel to stand.  12. Sit-and-reach test.  13. 6-minute walk test  14. Mobility Questionnaire  15. Cerebral Palsy Quality of Life Questionnaire for Children (CPP QOL-Child) and Cerebral Palsy Quality of Life questionnaire for Adolescents  16. Wong-Baker Faces Pain Rating Scale | Q:   - Children diagnosed with unilateral or   bilateral cerebral palsy (in functional severity Gross Motor Function Classification System levels I–III) | Physical health challenges |
| Lo (2018) | Reductions in behavioral problems and improvements in attention and behavioral rebulation. | 1. Self regulation  2. Attention regulation: conflict monitoring, orienting and alerting, total response time, and accuracy  3. Anxiety/depression, withdrawn, somatic complaints, attention problems, aggressive behavior, internalizing problems, externalizing problems, total problems score | 1. Counting Span Test.  1. Head-Toes-Knees-Shoulders task.  2. Attention Network Task.  3. CBCL Anxiety/depression, Withdrawn, Somatic Complains, Attention problems, Aggressive Behavior, Internalizing, Externalizing subscales and Total scales. | Q:   - Recruitment from eight primary schools or integrated family service centers from four Hong Kong districts ranked in the top five in terms of percentage of low-income population. - Children without developmental disorders, such as attention deficit hyperactivity disorder or autistic spectrum disorder. - Parents without psychosis, including schizophrenia and bipolar disorder.   Q´:   - Families in receipt of the Comprehensive Social Security Scheme (asset upper limit US$10.769 for four-person families) and families receiving the full rate School Textbook Assistance Scheme (monthly household income upper limit and asset upper limit for four-person families is US$2.577 and US$65.898, respectively). At risk subgroup analyses with families scoring > 100 in the Parenting Stress Index Short Form. | Socio-economic status |
| Webb (2018) | Improvements in mindfulness, other elements of self-regulation and HIV disease management. | 1. Mindfulness  2. Perceived stress  3. Coping strategies: rumination, distraction and problem-solving  4. Physical aggression  5. Current life satisfaction, illness-related anxiety and illness burden.  6. Cognitive self-regulation  7. Expressive attention  8. Medication adherence.  9. CD4 and HIV viral load (HIV VL) counts. | 1. MAAS  2. PSS.  3. Response Style Questionnaire  4. Aggression Scale  5. HIV Quality of Life scale  6. Stroop task: The Cognitive Assessment System Stroop task  Stroop task: The Color-Word Stroop  7. Emotion Stroop  8. Medication adherence measure ad-hoc  9. Medical records | Q:   - HIV infected adolescents receiving treatment at two urban clinics. Without significant cognitive, behavioral or psychiatric disorder that could interfere with group-based activities (determined by their clinical providers).   Q’:   - Having a current CD4 count above 200. | Physical health challenges |
| Lawler (2019) | Improvements in behavioral self-regulation and emotion-regulation. | 1. Selective attention and inhibitory control  2. Delay of gratification  3. Behavioral inhibitory control/effortful control and delay of gratification  4. Emotion regulation and “hot” executive function  5. Hyperactivity/inattention  6. Parent reported internalizing and externalizing symptoms. | 1. Color flanker task  2. Star delay task  3. Dinky toys task  4. Emotion-induction GNG task.  5. Strengths and Difficulties Questionnaire  6. MacArthur Health and Behavior Questionnaire | Q:   - Children from a database of Internationally Adopted children interested in research. - Parent report of diagnosis of fetal alcohol syndrome. Children without autism spectrum disorders or severe cognitive impairment. | Out of home placement |
| Lu (2019) | Reductions in social anxiety and suicide ideation. Improvements in mindfulness and self-esteem. | 1. Mindfulness  2. Social anxiety: fear of negative evaluation by peers, social avoidance and distress and total score.  3. Self-esteem.  4. Suicide ideation: positive ideation, negative suicide ideation and total score. | 1. MAAS  2. Social Anxiety Scale for Children  3. SES  4. Positive and Negative suicide Ideation | Q:   - Left-behind children attending to school, with no serious psychological problems that might interfere with the study (ensured by the teachers).   Q´:   - 54.0% with one parent absent and 46.0% with both parents absent. | Out of home placement/parental absence |
| Patton (2019) | Reductions in the growth of alcohol use. | 1. Alcohol use.  2. Alcohol related cognitions: positive expectancy and negative expectancy.  3. Drinking-refusal self-efficacy.  4. Impulsivity: reward drive.  5. Rash impulsiveness.  6. Socioeconomic background.  7. Mindfulness | 1. Alcohol Use Disorders Identification Test  2. Drinking Expectancy Questionnaire-Adolescent version.  3. Drinking Refusal Self-Efficacy Questionnaire-Revised Adolescent version  4. Shortened Sensitivity to Reward Scale.  5. Barratt Impulsiveness Scale – Brief  6. Family Affluence Scale-II  7. MAAS | Q:   - Students in grade 9 or 10 from six Australian schools. Most participants lived in medium affluence families and had Australian or European backgrounds.   Q´:   - 82.67% medium family affluence according to the Family Affluence Scale-II | Normative risk |
| Shomaker (2019b) | Reductions in perceived stress, food reward sensitivity, stress-eating, excess weight gain and body fat increases and improvements in executive functions. | 1. Perceived stress.  2. Food reward sensitivity.  3. Stress-eating.  4. Executive function.  5. BMI.  6. Percent body fat. | 1. PSS.  2. Behavioral task.  3. Total energy intake measured after the Trier Social Stress Test.  4. Behavior Rating Inventory of Executive Function - Inhibit, Shift, Emotional control, Initiate, Working memory, Plan/organize, Organization of materials and Monitor Scales.  4. National Institutes of Health Toolbox Flanker Inhibitory Control and Attention Test.  4. National Institutes of Health Toolbox List Sorting Working Memory Test.  5. Digital scale & stadiometer.  6. Air displacement plethysmography. | Q:   - Healthy adolescents at-risk for excess weight gain. In good general health, based upon a health history conducted with a parent and including no major medical problems (e.g. type 1 or 2 diabetes, musculoskeletal problems). Free of psychiatric symptoms that would impede compliance and necessitate treatment (e.g. suicidal behavior). Not takin medication affecting weight, mood, and/or eating (e.g., antidepressants, insulin sensitizers, stimulants).   Q’:   - BMI >=70^th^ percentile for age/sex or two biological parents with reported obesity (BMI>=30 kg/m^2^). | Physical health challenges |
| Vohra (2019) | Reductions in mental health symptoms and improvements in coping/resiliency. | 1. School problems, internalizing problems, emotional symptoms, personal adjustment (self-reported).  Externalizing problems, internalizing problems, school problems, behavioral symptoms, adaptive skills (teacher-reported)  Externalizing problems, internalizing problems, behavioral symptoms, adaptive skills (parent reported)  2. Stress levels.  3. Emotional regulation: Cognitive reappraisal and expressive suppression  4. Mindfulness | 1. Behavior Assessment System for Children, Second Edition  2. PSS.  3. Emotional Regulation Questionnaire  4. CAMM | Q:   - Residents of CASA House in Sherwood Park, able to communicate in and comprehend the English language, with a history of not responding to previous mental health interventions. Excluded if they had a diagnosis of psychosis. | Mental health challenges |
| *Note:* M = mean; SD = Standard Deviation; PTSD = Post Traumatic Stress Disorder; DSM = Diagnostic and Statistical Manual of Mental Disorders; SES= Socioeconomic status; MBT= Mindfulness-based terapy; BDI = Body mass index; PSS-10 = Perceived Stress Scale; STAI-C = Spielberger State-Trait Anxiety Inventory-Child version; SCL-90-R = Hopkins Symptom Checklist 90 Revised; SES = Rosenberg Self-Esteem Scale; CBCL = Child Behavior Checklist; DASS-21 = Depression Anxiety Stress Scales; EDE-Q = Eating Disorder Examination Questionnaire; CAMM = Child and Adolescent Mindfulness Measure; MAAS = Mindfulness Attention Awareness Scale; SCAS = Spence Children’s Anxiety Scale; PSQI = Pittsburgh Sleep Quality Index; T2D = Type 2 diabetes.  Studies corresponding to the same trial = ^1, 2, 3^ | | | | | |

| ***Table S2:*** Risk of bias summary of studies included in qualitative and quantitative analyses. | | | | | | | | |
| --- | --- | --- | --- | --- | --- | --- | --- | --- |
|  | Risk of bias items | | | | | | | |
| Study | 1 | 2 | 3 | 4 | 5 | 6 | 7 | 8 |
|  |  |  |  |  |  |  |  |  |
| Barnes (2001) | 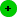 | 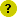 | 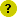 | 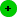 | 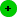 | 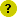 | 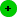 | 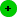 |
| Barnes (2003) | 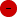 | 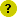 | 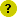 | 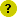 | 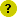 | 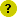 | 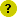 | 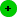 |
| Barnes (2004) | 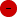 | 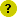 | 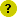 | 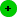 | 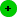 | 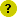 | 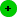 | 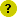 |
| Barnes (2004b) | 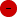 | 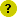 | 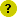 | 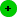 | 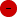 | 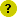 | 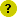 | 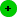 |
| Napoli (2005) | 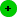 | 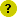 | 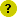 | 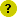 | 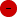 | 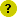 | 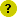 | 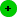 |
| Barnes (2008) | 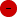 | 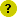 | 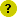 | 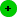 | 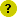 | 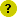 | 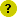 | 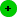 |
| Gordon (2008) | 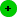 | 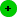 | 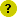 | 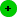 | 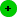 | 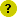 | 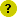 | 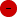 |
| Biegel (2009) | 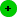 | 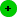 | 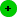 | 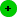 | 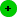 | 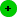 | 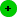 | 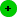 |
| Catani (2009) | 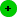 | 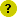 | 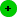 | 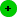 | 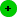 | 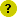 | 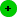 | 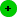 |
| Barnes (2010) | 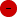 | 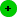 | 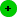 | 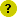 | 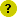 | 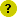 | 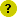 | 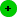 |
| Flook (2010) | 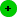 | 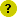 | 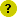 | 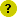 | 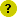 | 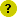 | 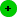 | 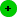 |
| Liehr (2010) | 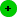 | 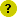 | 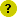 | 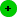 | 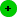 | 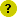 | 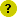 | 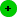 |
| Mendelson (2010) | 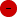 | 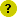 |  |  |  |  |  |  |
| Semple (2010) |  |  |  |  |  |  |  |  |
| Terjestam (2010) |  |  |  |  |  |  |  |  |
| Brown-Wright (2011), Gregoski (2011) |  |  |  |  |  |  |  |  |
| White (2012) |  |  |  |  |  |  |  |  |
| Evans-Chase (2013, 2015) |  |  |  |  |  |  |  |  |
| Leonard (2013) |  |  |  |  |  |  |  |  |
| Mano (2013) |  |  |  |  |  |  |  |  |
| Sibinga (2013) |  |  |  |  |  |  |  |  |
| Britton (2014) |  |  |  |  |  |  |  |  |
| Parker (2014) |  |  |  |  |  |  |  |  |
| Raes (2014) |  |  |  |  |  |  |  |  |
| Shirk (2014) |  |  |  |  |  |  |  |  |
| Sibinga (2014) |  |  |  |  |  |  |  |  |
| van de Weijer-Bergsma (2014) |  |  |  |  |  |  |  |  |
| Atkinson (2015) |  |  |  |  |  |  |  |  |
| Flook (2015) |  |  |  |  |  |  |  |  |
| Himelstein (2015) |  |  |  |  |  |  |  |  |
| Jee (2015) |  |  |  |  |  |  |  |  |
| Ricarte (2015) |  |  |  |  |  |  |  |  |
| Schonert-Reichl (2015) |  |  |  |  |  |  |  |  |
| Tan (2015) |  |  |  |  |  |  |  |  |
| Blake (2016, 2017a, 2017b,2018) |  |  |  |  |  |  |  |  |
| Bluth (2016) |  |  |  |  |  |  |  |  |
| Daly (2016) |  |  |  |  |  |  |  |  |
| Johnson (2016) |  |  |  |  |  |  |  |  |
| Poehlmann-Tynan (2016) |  |  |  |  |  |  |  |  |
| Quach (2016) |  |  |  |  |  |  |  |  |
| Sibinga (2016) |  |  |  |  |  |  |  |  |
| Felver (2017) |  |  |  |  |  |  |  |  |
| Johnson (2017) |  |  |  |  |  |  |  |  |
| Shomaker (2017, 2019a) |  |  |  |  |  |  |  |  |
| Mak (2018) |  |  |  |  |  |  |  |  |
| Lo (2018) |  |  |  |  |  |  |  |  |
| Webb (2018) |  |  |  |  |  |  |  |  |
| Lawler (2019) |  |  |  |  |  |  |  |  |
| Lu (2019) |  |  |  |  |  |  |  |  |
| Patton (2019) |  |  |  |  |  |  |  |  |
| Shomaker (2019b) |  |  |  |  |  |  |  |  |
| Vohra (2019) |  |  |  |  |  |  |  |  |
| *Note:* 1 = Sequence generation, 2 = Sequence concealment, 3 = Outcome assessment, 4 = Drop-outs, 5 = Intention-to-treat/missing data, 6 = Power analysis, 7 = Baseline imbalance, 8 = Diagnostic assessment. | | | | | | | | |
